# Conversational Agents: Goals, Technologies, Vision and Challenges

**DOI:** 10.3390/s21248448

**Published:** 2021-12-17

**Authors:** Merav Allouch, Amos Azaria, Rina Azoulay

**Affiliations:** 1 Computer Science Department, Ariel University, Ariel 40700, Israel; merav@g.jct.ac.il (M.A.); amos.azaria@ariel.ac.il (A.A.); 2 Department of Computer Science, Jerusalem College of Technology, Jerusalem 9116001, Israel

**Keywords:** smart environments, human–agent interaction, conversational agents

## Abstract

In recent years, conversational agents (CAs) have become ubiquitous and are a presence in our daily routines. It seems that the technology has finally ripened to advance the use of CAs in various domains, including commercial, healthcare, educational, political, industrial, and personal domains. In this study, the main areas in which CAs are successful are described along with the main technologies that enable the creation of CAs. Capable of conducting ongoing communication with humans, CAs are encountered in natural-language processing, deep learning, and technologies that integrate emotional aspects. The technologies used for the evaluation of CAs and publicly available datasets are outlined. In addition, several areas for future research are identified to address moral and security issues, given the current state of CA-related technological developments. The uniqueness of our review is that an overview of the concepts and building blocks of CAs is provided, and CAs are categorized according to their abilities and main application domains. In addition, the primary tools and datasets that may be useful for the development and evaluation of CAs of different categories are described. Finally, some thoughts and directions for future research are provided, and domains that may benefit from conversational agents are introduced.

## 1. Introduction

Conversational agents (CA) are agents that interact with users via written or spoken natural language. CAs accept as input natural language as speech, text, or video; in addition, they may receive input from several different sensors. CAs are required to process the input and provide relevant advice or feedback in a form of text or speech or by manipulating a physical or a virtual body. Some CAs are capable of taking specific actions either in the real world or in the virtual world. Most CAs use natural-language processing to understand and generate speech, and some may also have engagement and personalization abilities. The rapidly growing abilities introduced by modern machine learning techniques facilitate the development of CAs capable of carrying out meaningful conversations with humans, learning to generate better and more relevant responses, expanding their knowledge-base, and performing actions beneficial to their users.

Current technological development enables the increasing use of CAs in several domains, such as assistance agents in the educational domain and health system, customer support agents in the commercial domain, and influence bots in the political domain. Commercial CAs for personal use, such as Siri [1] of Apple, Meena [2] of Google, and Cortana [3] of Microsoft, are widely used around the world. The aim of our study was to outline the principles behind the development of CAs and to survey the main domains in which conversational agents are successfully used.

Several recent studies have been carried out over the last years on CAs and, in particular, on text-based CAs that are called chatbots (as defined in Section 2). Some studies concentrate on the technologies behind the development of CAs, and other studies examine their impact on people, i.e., the way people interact with them and perceive them.

Several recent reviews survey CA development and usage, at times referring to them as chatbots. Adamopoulou and Moussiades [4] provide a historical perspective of the chatbot development process, present a complete chatbot-categorization system, and analyze the two main approaches in chatbot development: pattern matching and machine learning. They mention two limitations of the current generation chatbots in understanding and producing natural speech, and they also point out that today’s technology aims to build chatbots that can learn to talk but that cannot learn to think.

In another study, Adamopoulou and Moussiades [5] present an overview of the evolution of the international community’s interest in chatbots and discuss the motivations that drive the use of chatbots and their usefulness in a variety of areas. They clarify the technological concepts and classify them based on various criteria, such as the area of knowledge and the need they serve. Furthermore, they present the general architecture of modern chatbots while also mentioning the main platforms they were created for. In another study, Nuruzzaman et al. [6] present a survey on commonly used chatbots and the underlying techniques. They focus on response-generating chatbots. In this category, the various response models can be categorized into four groups: template-based, generative, retrieval-based, and search engines. They compare the 11 most-popular chatbot application systems and present the similarities, differences, and limitations. They conclude that despite recent technological advances, chatbots conversing in a human-like manner are still hard to achieve.

Another survey concentrating on the technologies used by CAs is that of Borah et al. [7]. They describe the overall architecture of CAs, concentrating on the machine learning layer and analyze the recent development of text-based CAs. Chen et al. [8] describe the technology behind CAs and dialogue systems in real-world applications and discuss the effect of recent advances in deep learning on CA development. They emphasize that “big data” available from conversations on social media can be useful in building data-driven, open-domain CAs capable of responding to nearly any query. They further state that deep learning technologies can be used to leverage the massive amount of data to advance CAs from different perspectives. Gao et al. [9] concentrate on deep learning based CAs. They group the conversational agents into three categories: question-answering agents, task-oriented dialogue agents, and chatbots. For each category, they present a review of state-of-the-art neural approaches, draw the connection between neural and traditional approaches, and discuss the progress that has been made and challenges still being faced using specific systems and models as case studies.

Diederich et al. [10] review 36 studies on CAs in information systems (IS). They classify the literature along five dimensions. Three dimensions are related to CAs: the mode of communication, the context, and embodiment; and the other two dimensions are related to IS: the theory type and the research method. Wolff et al. [11] define a set of criteria to categorize chatbot applications. They review 52 articles describing chatbots. Most of the articles focus on customer-support chatbots, e.g., chatbots used to acquire information on specific services or products. In this article, we provide an overview of the concepts and building blocks of CAs and categorized them according to their abilities as well as the main domains of application. We emphasize the challenges and issues related to CA development for each domain while describing the tools and datasets useful for the development and evaluation of CAs of different categories. Finally, we provide some thoughts and directions for future studies and introduce domains that may benefit from conversational agents. For each of the topics in this survey, we focus on studies from the recent five years, though we also include earlier seminal studies as well as classical evaluation methods. In addition, the datasets provided in Section 8 include any relevant dataset that we found and are not limited to recent datasets.

The remainder of this article is organized as follows. Section 2 provides the terms and concepts used in the domain of conversational agents and defines the terms used in this study. Section 3 describes the design components of primary CA types. Section 4 and Section 5 survey the main technologies used for conversational software development, including machine learning (ML) methods and advanced technologies that enhance emotional abilities. Section 6 surveys recent CA applications, including personal assistants, healthcare agents, e-learning agents, and customer-support chatbots. The second part of this review focuses on technological issues. Section 7 and Section 8 review commonly used datasets for CA development and testing and the technologies used to evaluate CAs. Finally, Section 9 concludes by providing ideas and directions for future developments.

## 2. Related Definitions and Terms

Conversational agents are highly referenced in the literature by numerous sources, including research articles, industry documentations, and internet blogs. Unfortunately, there exist inconsistencies in the references with respect to several central concepts related to conversational agents. Therefore, the aim of this section is to improve clarity, by providing definitions for the main relevant concepts currently in use, such as conversational agents, dialogue systems, chatbots, and virtual assistants.

It was observed that there are two terms that are sometimes used interchangeably: the term *conversational agent* and the term *chatbot*. There have been several attempts to define the distinction between the two terms. According to Vishnoi’s definition [12], chatbots are software components that are designed to respond to human statements with a specific set of predefined replies. However, conversational agents are more contextual than chatbots and use more-advanced technologies such as deep learning methods and natural language understanding (NLU).

According to Nuseibeh [13], conversational agents are all types of software programs that interpret and respond to statements made by users in natural language. Chatbots, according to this definition, are a type of CA designed to simulate conversations with human users. Other types of CAs are programs designed to perform a particular goal, such as vacation planning and booking. CAs of this type are called *goal-oriented conversational agents*.

Radziwill and Benton [14] define conversational agents as software systems that mimic interactions with real people. They define chatbots as CAs that are implemented using a text-based interface.

Hussain et al. [15] classify chatbots into two main categories: task-oriented chatbots and non-task-oriented chatbots. According to Hussain et al., task-oriented chatbots are designed to accomplish specific goals such as ordering a pizza, guiding a user on social media, etc. The non-task-oriented chatbots for entertainment converse with users in an open domain. Masche and Le [16] categorize conversational systems into chatbots and dialogue systems. According to their definition, chatbots are systems mainly based on pattern matching, while dialogue systems are based on theoretically motivated techniques that enable conversations. Nimavat and Champaneria [17] distinguish between four criteria that can be used to classify chatbots: the knowledge domain, the type of service provided, the chatbot goal, and the the response-generation method. They define conversational bots as bots that talk to the user like another human being, in an open domain. It is worth noting that due to the ambiguity in the related terms and definitions, and the lack of a commonly agreed upon standard on the meaning of chatbot, the Alexa prize competition, set up with the goal of furthering conversational AI, uses the term *socialbot* to describe the conversational agents. These agents are intended to interact on a range of open-domain conversational topics [18].

In this review, our own definition for CA is provided, which is built upon the definitions provided in previous studies. To properly define CA, the more general concept of dialogue systems is introduced first. A *dialogue system* is a human–computer interaction system that uses natural language to communicate with the user. A *conversational agent* is a dialogue system that can also understand and generate natural language content, using text, voice, or hand gestures, such as sign language. Thus, to be categorized as CA, the condition is, according to our definition, being able to understand and produce *sentences* in natural language. As a result, a CA is required to handle natural language that is not limited to a predetermined set of words (e.g., only numbers or a set of keywords) or a limited sentence structure.

The following examples cannot be considered CAs: (a) An interactive voice response (IVR) system in which the user is instructed to press a number on a keypad or say a specific word in order to advance to the next menu (e.g., “Press or Say 1 for English”) is not considered a CA, since the user response does not include natural language *sentences*. (b) An embedded system in which a user provides voice commands (e.g., ”Turn on the lights” or ”Set the temperature to 25 degrees”) and the system executes them without invoking any natural language response.

**Figure 1:**
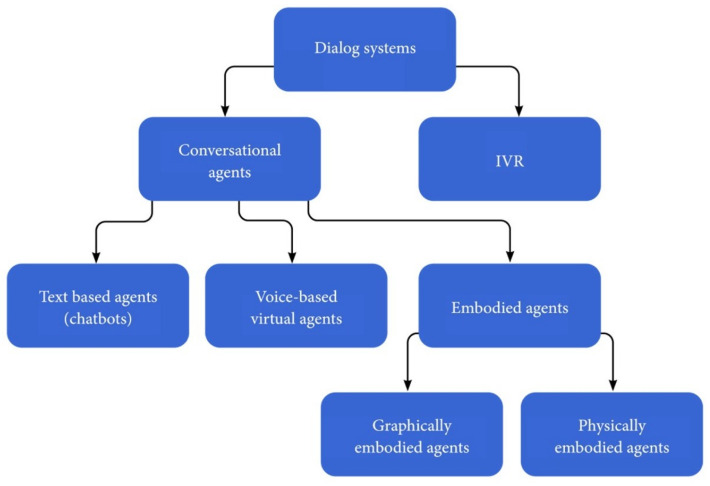
Conversational agents and chatbots: the definitions used in this article.

There are different criteria for categorizing CAs: the mode of communication, the action capabilities, and the domain/application in which the CA operates. First, our definition of conversational agents is refined according to the mode of communication between the CA and the human user. Here, a *chatbot* is defined as a CA that interacts with the user only by text and not by any other means of communication, for example, the ELIZA chatbot [19], or chatbots available on service platforms, such as banks, booking, and other e-commerce domains. Voice-based virtual agents are CAs that interact with the users by voice, for example, Siri, Google Now, Cortana, etc. Graphically embodied agents are virtual agents that have a virtual body as well as voice-understanding and speech-generation abilities. Their virtual body enables them to provide an additional means of communication through gestures. Finally, physical-based embodied agents are CAs that have a physical body, such as social robots, e.g., JIBO [20]. Both graphical and physical agents are called embodied CAs (ECAs). The above definitions are used throughout this article and are summarized in Figure 1.

**Figure 2:**
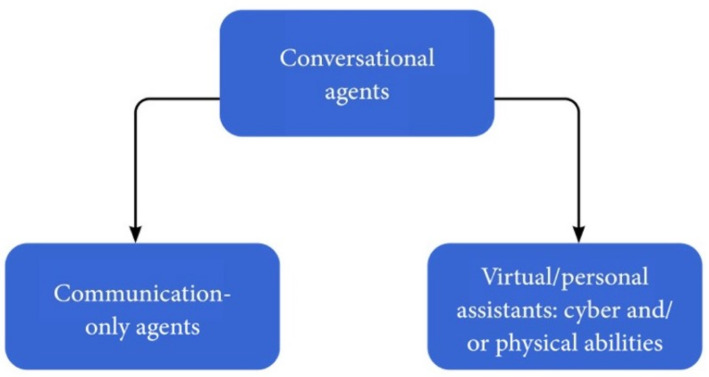
Conversational-agent classification according to action capabilities.

CAs can also be classified according to their effector capabilities and actions. Communication-only agents merely communicate with a user and do not execute any action, e.g., ELIZA [19], Cleverbot [21,22] or CAs used only to answer questions. Other CAs, known as virtual or personal assistants, e.g., Alexa [23], are capable of executing physical or virtual actions, such as turning on an AC or booking a flight (see Figure 2).

**Figure 3:**
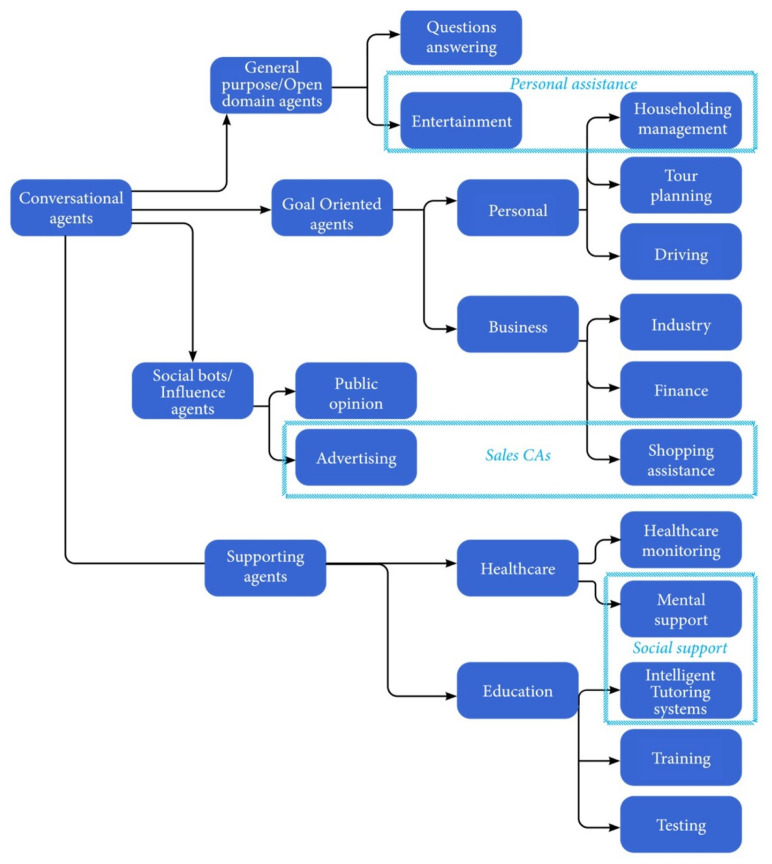
Conversational-agent applications.

Finally, CAs can be classified according to the application: (a) Open domain/general purpose CAs are mainly used to answer questions in various domains or in entertainment and are mostly communication-only agents. (b) Goal-oriented CAs assist users in completing tasks requiring multiple steps and decisions. Goal-oriented CAs are also task-oriented dialogue systems [24] and are referred to as taskbots according to the Alexa Prize competition [25]. These agents may be used both in the business domain or as personal assistants. In the business domain, they operate as customer-service and sales representatives. As personal support agents, they can assist the user in particular tasks, such as driving, vacation planning, or trip management. (c) Social-supporting agents can support patients in medical conditions or support students in the learning process. (d) Social-network bots, also known as influence agents, are intelligent CAs acting in social media to advertise a product or to influence opinions (see Figure 3). The rest of the article uses the terms defined in Figure 1 while considering various CA applications, as detailed in Figure 3. A detailed survey on CA usage in various domains is provided in Section 6.

## 3. CA’s Design Issues

This section describes the different components related to CA design. CA design is divided into four classes: text components for chatbots; CA components related to voice-based virtual agents; physical-related components for goal-oriented CAs or for embodied agents; and task-performance components for goal oriented CAs. For each of the four classes, the general goal is provided, the main components are detailed, and the relations between these components are described.

### 3.1. Text Related Components

The two main abilities required of CAs are the ability to logically understand the user’s utterance and the ability to correctly reply to it. Overcoming these challenges require research in the fields of natural-language processing (NLP), information retrieval (IR), and machine learning (ML) [9].

Text-related components are used by most CAs, including embodied CAs and voice-based CAs, since voice-based virtual agents usually translate human speech to text, analyze the text, generate text responses, and then produce the speech signals. Therefore, in our design description, text-related components are discussed first.

CAs are commonly partitioned into components based on a pipeline determined by the order in which the component is used [26,27]. The most-common components are

- The natural-language-understanding (NLU) component: interprets the words into an internal computer language, called a logical form, which represents the meaning of the text.
- The dialogue manager component: receives the logical form and decides on how to respond. The dialogue manager may also include a module that assists with long-term conversations.
- The natural-language-generation (NLG) component: converts the answer into a text sequence in natural human language.

**Figure 4:**
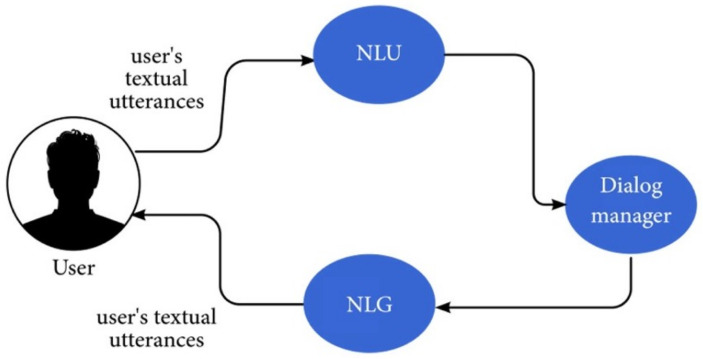
The textual components of CAs.

A schematic description of the textual processing components is provided in Figure 4.

Masche and Le [16] use a similar categorization, with an additional preprocessing component. They provide an alternative hierarchical approach to define text-related components by dividing the components into those responsible for text understanding, text processing, and text producing, as defined by Stoner et al. [28], as follows:

- Responder—the interface between the user and the CA: transfers and monitors the inputs and the outputs.
- Classifier—the interface between the responder and the graphmaster: normalizes and filters user inputs and processes the graphmaster output.
- Graphmaster—the brain behind the CA: manages the high-level algorithms.

According to this approach, the responder component includes parts from both NLU and NLG, while the dialogue manager component has parts from both the classifier and the graphmaster.

Abdul-Kader et al. [29] survey the techniques used to design CAs and describe the main techniques used by pattern-matching-based CAs, which are: (a) Parsing: manipulation of the input text using NLU functionality. (b) Pattern matching: analyzing user input and collecting relevant data, especially used by question-answering systems. (c) Chat script: used when no matches occur. (d) History database: used to enable the chatbot to remember previous conversations. (e) Markov Chain: enables probabilistic-based responses of chatbots.

Ramesh et al. [30] describe various approaches to design and build chatbots. Ahmad et al. [31] provide some examples of chatbots, describe their design, and provide a description of the most-popular techniques used by chatbot developers. Diederich et al. [32] analyze 51 CA platforms to develop a taxonomy that would allow the identification of platform archetypes in CA design. The taxonomy consists of eleven dimensions and three archetypes, which can be used by practitioners in the design stages of CA. Lokman and Ameedeen [33] categorize modern chatbot design into the following elements: domain knowledge, response generation (retrieval or generative), text processing (vector embedding or Latin alphabet), and machine learning (ML) (mostly using neural networks). The various components described in this section enable the creation of CAs that are able to communicate with humans through an appropriate textual interface. In the next section, these technologies are also used for other types of CAs, such as voice-based CAs.

### 3.2. Voice-Related Components

Voice-based virtual agents are CAs that communicate with humans using speech. The process used by CAs usually includes: translating the sound waves into text, understanding the text, producing a text response for the user, and translating the text response to the sound produced by the computer or by the robot. The steps of understanding the text and producing an answer usually rely on the text-related components described above, but there are additional components, such as voice-based virtual agents related to audio analysis and audio production. A voice-based virtual agent may extract additional non-verbal information from the user audio, such as the user’s emotional state, e.g., whether the user is being sarcastic, dramatic, decisive, or trying to deceive the system. Some works have also used non-verbal cues to detect whether a user is trying to correct previously made statements [34]. The components responsible for additional voice-based capabilities include:

- An automatic-speech-recognition (ASR) component (speech to text): converts the audio stream to a text representation.
- Non-verbal-information-extraction component: extracts relevant non-verbal information from the audio, such as observing the user’s emotional state or understanding the urgency.
- Text-to-speech component: synthesizes the output waveform that is sent to the speakers.

**Figure 5:**
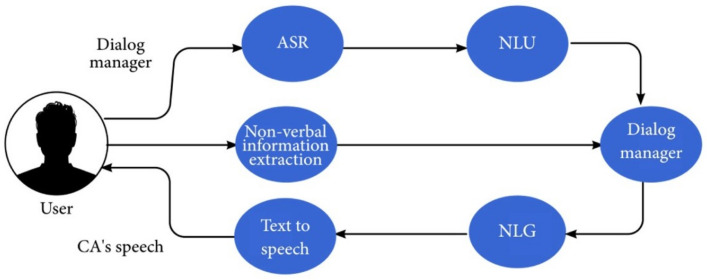
The main voice-based components of CAs.

The main components of the audio-process components are described in Figure 5.

Additional information on the capabilities and components of speech-based CAs is described by Saund [35]. Benzeguiba et al. [36] review ASR challenges and technologies, and Yu and Deng [37] provide a complete overview on modern ASR technologies with an emphasis on the deep-learning methods adopted in ASR.

### 3.3. Physical-Related Components

Physical embedded CAs, which obtain visual input from the user, benefit from the ability to understand physical-related gestures, such as body language and facial expressions. In addition, embodied CAs (ECAs) can use facial expressions and body gestures in their reactions.

Sign languages are complete languages that use only physical gestures to communicate. These languages may be used by CAs designed to communicate and/or tutor deaf users. Next, the main components in building an agent with these capabilities are described while referring the reader to articles reviewing this field.

Sadeghipour and Kopp [38] describe an overall model for cognitive processes of embodied perception and generation. According to them, the main components for physical agent–human communication are as follows:

- Perception component: receives visual movements and preprocesses them. The preprocessing pipeline consists of four submodules: (1) The body correspondence solver is responsible for performing required operations (such as rotation and scaling) on the observations. (2) The sensory memory receives the transformed positions and buffers them in chronological order. (3) The working memory holds a continuous trajectory for each hand through agent-centric space. (4) The segmenter submodule decomposes the received trajectory into movement segments called guiding strokes.
- The shared-knowledge component is responsible for the representation of motor knowledge. This component consists of a hierarchical structure, starting with the form of single-gesture performances in terms of movement trajectories and leading into less-contextualized motor levels and then toward more context. The motor-representation hierarchy consists of three levels: motor commands, motor programs, and motor schemas.
- The gesture-generator component is invoked by a prior decision to express an intention through a gesture. This component may also be used by a virtual agent that is built on a motor-control engine.

**Figure 6:**
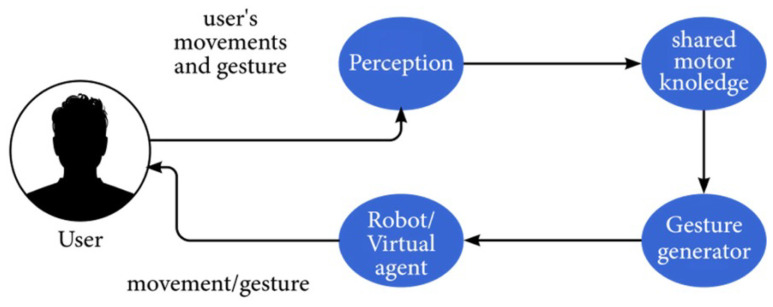
The main components of a physical-based embodied CA.

The main components of the physical-based, embodied CA are described in Figure 6. Krishnaswamy et al. [39]. provide a review on sign languages and gesture interpretation and generation. Homburg et al. [40] describe the process of sign-language (SL) translation, including SL recognition and SL generation. Singh et al. [41] detail the process of recognizing and interpreting the Indian sign language. Finally, Beck et al. [42] study the generation of emotional body language to be displayed by humanoid robots.

### 3.4. Task-Related Components

Goal-oriented CAs assist users in completing tasks requiring multiple steps and decisions, such as CAs booking vacations and planning trips. Goal-oriented CAs may use the text-related and voice-related components described above, in addition to task-related components. Task-related components are special components that handle task-related planning and learn challenges for the successful execution of the required goal. Previous studies on goal-oriented CAs [43,44] describe the processes followed by a conventional goal-oriented CA. This process includes the phases of text understanding, state estimation, dialogue policy, and text generation. The additional task-related components are defined as follows:

- State tracker: estimates the state of the user’s goal by tracking the information across all turns of the dialogue.
- Policy manager: determines the next set of actions to help reach that goal. The policy manager uses the goal-related information from the state tracker and may communicate with the dialogue manager.
- Action manager: performs the required cyber actions (e.g., hotel reservations, food ordering, and flight booking) and/or the required physical actions to successfully fulfill the user requests.

**Figure 7:**
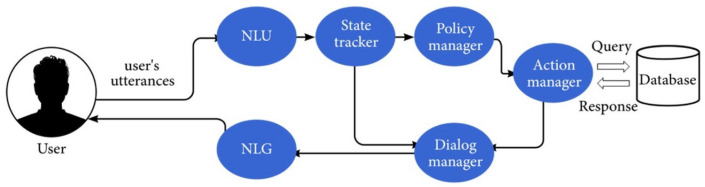
The main components of a goal-oriented CA.

The schematic description of the task-related components is provided in Figure 7, and an overview of the technologies behind goal oriented CAs is provided in Section 4.5.

## 4. Technologies behind CA Components

In this section, the technologies behind the CA components presented in Section 3 are described in further detail, detailed examples are provided for the physical components, and the implementation of the technologies in recent CA systems are discussed.

### 4.1. Natural Language Understanding

Natural language understanding (NLU) typically refers to extracting structured semantic knowledge from text. NLU tasks mainly include tokenizing the text, normalizing it, recognizing the text entities, and performing dependency or constituency parsing. The traditional NLU stack is based on the following five components: phonology, morphology, syntax, semantics, and reasoning [45].

In particular, morphological analysis or parsing can be viewed as resolving natural-language ambiguity at different levels by mapping a natural language sentence to a series of human-defined, unambiguous, symbolic representations, such as part-of-speech (POS) tags, context-free grammar, and first-order predicate calculus. NLU includes the following sub areas: resolution, discourse analysis, machine translation, morphological segmentation, named-entity recognition, POS tagging, and more [27]. For a review on natural language understanding, the reader is referred to the survey of Navigli [46], in which several NLU approaches and modes are reviewed, including explicit versus implicit learning, representation of words and semantics, and a vision on what machines are expected to understand.

In the remainder of this section, the focus is on studies that use NLU for CA development. Initially, CAs using classical NLU technologies are described. Next, CAs using a parser as their NLU component are described. To conclude, recent CAs that use advanced technologies for NLU are described.

A classical approach for designing chatbots is the pattern-matching approach, in which the CA matches the user input with a pattern and chooses the most-suitable response stored in its predefined text corpus. One example of a CA that is based solely on simple pattern matching is ELIZA [19]. Over the years, several studies have developed additional rules and corpora to develop more-adaptive and advanced CAs. Inui et al. [47] use a linguistic corpus to design a CA interface. The dialogue corpus is based on a series of dialogues, and NLU is achieved by adopting corpus-based methods like the stochastic model, the n-gram model, keyword matching, and structural matching.

ALICE [48] is a chatbot based on AIML [49], an XML-based language designed to create chatbots based on pattern matching. ALICE won the Loebner Prize as “the most human computer” at the annual Turing Test contests of 2000, 2001, and 2004. ALICE answers the user’s query by using its pattern-matching engine, which searches for a lexical correspondence between the user’s query and the chatbot’s patterns.

Agostaro et al. [50] outline the limitations of the pattern-matching approach. Pattern matching may fail to answer the user query when the query is composed of words that do not match any pattern. Therefore, when the query is grammatically incorrect, the pattern-matching mechanism will fail. To overcome these limitations, Agostaro et al. developed LSA-bot [50], which is a chatbot based on latent semantic analysis (LSA). LSA applies statistical computations to a large corpus of text to extract and represent the meaning of words. LSA-bot uses LSA to map its knowledge base into a conceptual space. The user input is mapped into the same conceptual space, allowing LSA-bot to find an appropriate response.

The informal response interactive system (IRIS) chatbot, developed by Banchs and Li [51], uses a large database of dialogues to provide candidate responses to a given user utterance. The IRIS response-selection process chooses the candidate utterances using two scores. The first score is determined by the cosine similarities between the current user input vector and all single utterances stored in the database. The second score is determined by the cosine similarity between the current vector dialogue and the dialogue history of the user. The two scores are combined using a log-linear scheme. The IRIS randomly selects one of the top-ranked utterances as its response.

A context-free-grammar (CFG) parser [52] is often used by CAs for NLU. A CFG parser builds a constituency parse tree from the given user utterance based on a grammar, which is composed of parsing rules. A more generalized CFG, which is more suitable for solving ambiguity, is the probabilistic CFG (PCFG) [53,54]. In a PCFG parser, each rule in the grammar is associated with some probability. A PCFG parser outputs the parse tree with the highest probability.

Azaria et al. [55] present LIA, an agent that uses a combinatory categorial grammar (CCG) parser as its NLU component. The parser maps the commands, which are given in natural language, to logical forms, which contain functions and concepts that can later be executed by the dialogue manager. CCGs benefit from being more expressive than CFGs as they can represent the long-range dependencies appearing in some sentences (e.g., relative clauses), which cannot be expressed using CFGs. Recent ML methods and word-embedding methods are widely adapted to achieve NLU components with higher performance. Rasa NLU and Rasa Core [56] are open-source Python libraries for building conversational software. Rasa NLU allows the use of a predefined pipeline for the NLU process.

Recent ML methods and word embedding methods are widely adapted for achieving NLU components with higher performance. Rasa NLU and Rasa Core [56] are open-source Python libraries for building conversational software.

Rasa NLU allows the use of a predefined pipline for the NLU process. Their recommended pipeline process starts by tokenizing the user input, followed by the conversion of each token to a GloVe embedding vector [57]. Then, a multiclass support vector machine (SVM) [58] is used for deciding which action to take. Custom entities are recognized using a conditional random field [59].

ConvLab-2 [24], which is an open-source toolkit for building goal oriented CAs, provides three NLU models: a semantic tuple classifier, a multi-intent language understanding model [60], and a fine-tuned BERT- [61] based NLU model with the ability of intent classification and slot tagging.

### 4.2. The Dialogue Manager

Given the input text, the next step in the CA’s pipeline is to manage the dialogue with the user. The *dialogue-manager* component is responsible for two main tasks: *Dialogue modeling*: keeps track of the state of the dialogue and *Dialogue control*: decides on the next system action [62].

Harms et al. [63] review the state-of-the-art commercial and research tools available for CA dialogue management. They divide the management approaches into two types: handcrafted-rule-based approaches and probabilistic (data-driven) approaches. The handcrafted dialogue manager defines the state and the control of the system by a set of rules that are defined by developers and experts, while the probabilistic dialogue manager learns the rules from actual conversations.

The studies described next concentrate on dialogue managers, including handcraft-rule-based systems and probabilistic-based systems. Handcraft rule-based management systems may be based on a planning algorithm or a pattern-matching based approach. Nguyen and Wobcke [64] propose a planning-based approach for developing a personal-assistant CA. In their approach, the dialogue manager has a set of plans, which can be divided into four groups: conversational-act determination and domain-task classification, intention identification, task processing, and response generation.

CommandTalk is a spoken-language interface for a battlefield military simulator [65,66]. It manages the representation of linguistic context, interprets user utterances within that context, and plans system responses. The CommandTalk dialogue manager uses a dialogue stack, a recovery mechanism for the stack, reference mechanisms, as well as finite state machines.

The MindMeld Conversational AI platform [67] is a platform designed for building conversational assistants. It uses pattern-matching rules to determine the dialogue state, and, based on this state and the predefined business logic, the CA performs the required task (or response) related to this state.

The Bottery CA creation platform [68] consists of four components: a set of states, a blackboard-style memory, an optional set of global transitions to allow the agent to switch from state to state, and an optional grammar used by the agent to generate the final outputs of the CAs. The Bottery syntax can be simply expressed by using structured JSON and can be extended by using imperative JavaScript code. The Bottery conversation management is performed by a finite state machine, which is displayed as a graph.

We proceed by describing probabilistic-based dialogue-management schemes. Google DialogFlow [69] is a framework for composing CAs. The Google dialogue manager considers the intent or motivation extracted from the user conversation to determine the appropriate action. Another commercial CA framework is Microsoft LUIS [70], a cloud-based conversational AI service that uses ML to understand the conversation to extract relevant information. LUIS can assist developers, who are unfamiliar with ML methods, to create their own cloud-based ML models specific to the application domain. Herderson et al. [71] present a word-based approach to dialogue state tracking using recurrent neural networks (RNNs). The model is capable of generalizing to unseen dialogue states’ hypotheses. For long-term effects of the conversation, dialogue managers consider the conversation as a Markov decision process (MDP) and choose their responses by using RL methods. Singh et al. [72] suggest using RL for goal-oriented dialogue management.

Li et al. [73] suggest applying DRL to model future rewards in CAs. The agent’s reward is determined according to three useful properties: informativity (non-repetitive turns), coherence, and ease of answering. The dialogue manager of the ensemble-based CA developed by Serban et al. [74] for the Amazon Alexa Prize competition utilizes an ensemble of NLG and retrieval models, including template-based models, bag-of-words models, sequence-to-sequence (seq2seq) neural networks, and latent-variable neural=network models. Their dialogue manager is trained to select an appropriate response by applying RL. The training was carried out on crowdsourced data as well as on real-world-user-interactions data.

### 4.3. Natural Language Generation

The NLG component translates the CA’s representation of the response to natural language. NLG is defined by Reiter and Dale [75] as a subfield of AI and computational linguistics that is concerned with producing understandable texts in some human language from some underlying non-linguistic representation of information. Gatt and Krahmer [76] provide a recent survey on state-of-the-art NLG research, focusing on data-to-text generation. They discuss NLG architectures and approaches and highlight several new developments. In addition, they review the challenges of NLG evaluation and show the relationships between different evaluation methods.

NLG can be performed by template-based systems, which map the non-linguistic input directly to the linguistic surface structure without intermediate representations. Van Dimter et al. [77] describe several template-based systems and compare them to other NLG systems in terms of their potential for performing NLG tasks. They claim that template-based systems can, in principle, perform all NLG tasks in a linguistically well-founded way.

Several recent CAs use deep neural networks (DNNs) to perform the natural language-generation task. Wen et al. [78] present a statistical language generator based on a semantically controlled long-short-term-memory (LSTM) structure. The LSTM generator is trained on unaligned data by jointly optimizing sentence planning and surface realization. Variations in natural-language output are obtained by randomly sampling the network output.

Tran et al. [79] present a semantic component, called an aggregator, which can be integrated into an existing RNN encoder–decoder architecture, to improve NLG performance. The proposed component consists of an aligner and a refiner. The aligner is a component that computes the attention over the encoded input information, while the refiner is a gating mechanism stacked over the attentive aligner to further select and aggregate the semantic elements.

Jeraska et al. [80] focus on language-generation models with inputs structured for meaning representation to describe a single dialogue act with a list of key concepts that need to be conveyed to the user. They present a neural ensemble encoder–decoder model for generating natural utterances from the meaning representations.

Dusek et al. [81] assess the capabilities of recent seq2seq data-driven NLG systems, which can be trained on pairs of sequences, without the need for fine-grained semantic alignments. These pairs of sequences are composed of meaning representations, which are the output of the dialogue manager and the corresponding natural-language texts. They find that seq2seq NLG systems generally score high in terms of word-overlap metrics and human evaluations of naturalness but often fail to correctly express a given meaning or representation if they lack a strong semantic-control mechanism during decoding. Moreover, they can be outperformed by hand-engineered systems in terms of the quality, complexity, and diversity of outputs.

### 4.4. End to End Models

A popular end-to-end technique used by CAs is based on sequence-to-sequence learning models. These models convert sequences from one domain into sequences in another domain. Sequence-to-sequence models are widely used in different domains, such as machine translation, text summarization, speech to text conversion, image-caption generation, and automated answer generation.

Sordoni et al. [82] present a sequence-to-sequence-based chatbot trained end-to-end on large quantities of unstructured Twitter conversations. A neural-network architecture was used to address sparsity issues that arise when integrating contextual information with classic statistical models, allowing the system to take into account previous dialogue utterances. They extended the recurrent-neural-network language model [83] and proposed a set of conditional language models in which past utterances are encoded in a continuous context vector to help generate the response.

Li et al. [84] propose a method for defining the sequence-to-sequence objective function. They proposed using MMI, a measurement of the mutual dependence between inputs and outputs, as the objective function for the generated conversational responses. They also present practical strategies for neural generation models that use MMI as the objective function. The experimental results demonstrate that the proposed MMI models produce more diverse, interesting, and appropriate responses, yielding substantial gains in BLEU scores and in human evaluations.

Serban et al. [85] investigate the task of building open-domain CAs based on large dialogue corpora using generative models. Generative models produce responses that are generated word-by-word, opening the possibility for realistic, flexible interactions. In their model, a dialogue is considered as a sequence of utterances that, in turn, are sequences of tokens. They extend the hierarchical recurrent encoder–decoder (HRED) neural network to the dialogue domain. Their experiments demonstrate that the hierarchical recurrent-neural-network generative model outperforms both n-gram-based models and baseline neural-network models in the task of modeling utterances and speech acts. In addition, they show that the performance of their system can be improved by bootstrapping the learning from a larger question–answer pair corpus and from pretrained word embeddings.

Some studies concentrate on seq2seq learning for question-answering chatbots. He et al. [86] suggest a model based on sequence-to-sequence learning for a question-answering chatbot, which can answer complex questions in a natural manner. The model incorporates copying and retrieving mechanisms in a bi-directional RNN. The semantic units in the answers are dynamically predicted from the vocabulary, copied from the given question, and/or retrieved from the corresponding knowledge base.

Qiu et al. [87] present a hybrid open-domain question-and-answer chatbot that combines information retrieval and seq2seq models. Information retrieval methods are used to retrieve a set of question/answer pairs based on a chat log of an online customer service. Then, the seq2seq model is used to rank the candidate answers. If the score of the top candidate answer is above a predefined threshold, it is considered to be the answer; otherwise, the answer is generated by the seq2seq model. Similarly, Ghazvininejad et al. [88] present a general data-driven and knowledge-grounded CA. They condition the CA responses not only on the conversation history but also on external facts through multi-task learning. This makes the CA versatile and applicable to an open-domain setting.

End-to-end models can also be useful in goal-oriented CA developments. Ham et al. [89] describe the use of end-to-end models for goal-oriented CAs, which need to integrate external systems to provide an explanation for the particular responses. They present an end-to-end monolithic neural model that learns to follow the core steps in the dialogue-management pipeline. The model outputs all the intermediate results in the dialogue-management pipeline to enable integration with the external system and to interpret why the system generates a particular response.

Kim [90] presents an end-to-end document-grounded, goal-oriented CA that utilizes a pretrained language model with an encoder–decoder structure. The encoder solves both the knowledge-seeking turn-detection task and the knowledge-selection task; the decoder solves the response-generation task.

Das et al. [91] suggest using DRL to learn the policies of goal-oriented CAs to answer visual questions. They pose a cooperative dialogue between two CAs communicating by natural language. The dialogue involves two collaborative CAs; one CA sees the image; and the second CA asks the first one questions about the image. DRL is used for learning the policies of these agents during the multi-round dialogue. As a result, the two trained CAs invent their own communication protocol without any human supervision.

### 4.5. Technologies Specific to Goal-Oriented CAs

In the development of goal-oriented CAs, there are additional challenges due to the need to combine both the dialogue handling and the task-performance management. Several ML-based technologies are commonly used to handle these challenges.

Zhang et al. [92] review the recent advances in goal-oriented CAs and discuss three critical topics: data efficiency, multi-turn dynamics, and knowledge integration. They also review the recent progress on task-oriented dialogue evaluation and widely used corpora, and they conclude by discussing some future trends for task-oriented CAs.

Zhao and Eskenazi [43] discuss the limitations of the conventional goal-oriented CA pipeline and suggest an alternative end-to-end task-oriented dialogue-management framework. In their framework, the state tracker is an LSTM-based classifier that inputs a dialogue history and predicts the slot-value of the latest question. The policy manager is implemented by a deep recurrent Q-network (DRQN) that controls the next verbal action. This framework enables the creation of a CA, which can interface with a relational database and learn policies for both language understanding and dialogue strategies.

Noroozi et al. [44] present a fast-schema-guided tracker (FastSGT), which is a BERT-based model for state tracking in goal-oriented CAs. FastSGT enables switching between services and accepting the values offered by the system during the dialogue. Finally, an attention-based projection is suggested to better model the encoded utterances.

Kim et al. [93] propose a two-step ANN-based dialogue-state tracker, which is composed of an informativeness classifier and a neural tracker. The informative CNN-based classifier filters out non-informative utterances, and the neural tracker estimates dialogue states from the remaining informative utterances.

Mrksic et al. [94] consider the issue of developing a state tracker for goal-oriented CAs. They consider the difficulty of scaling the state tracker to large and complex dialogue domains because of the dependency on large training sets. They propose a neural-belief-tracking (NBT) framework that uses pretrained word embeddings to learn the distribution of user contexts.

Su et al. [95] estimate the task success by inspecting the dialogue as it evolves, by utilizing RNNs and CNNs. Their experiments demonstrate that both RNNs and CNNs can accurately estimate when substantial training data are available, though RNNs are more robust when training data are limited. Many goal-oriented CAs are trained on available goal-oriented datasets (see Section 8.3 for more details on such datasets). Other goal-oriented CAs are trained on human users. While such training may yield richer dialogues, it is more expensive.

Liu and Lane [96] address the challenges of building a reliable user simulator to train a goal-oriented CA by simulating the dialogues between two agents. Initially, a basic conversational agent and a basic-user simulator are trained on dialogue corpora through supervised learning, and then their abilities are improved by allowing them to conduct task-oriented dialogues while iteratively improving the policies using DRL.

## 5. Human-Related Issues

In addition to the technical issues of natural language understanding and generation, good conversational agents should be aware of human characteristics, observe user emotions, provide empathy in their responses, and engage the user.

According to Clark et al. [97], humans perceive the communication with CA as a means to achieve functional goals. In their study, Clark et al. present the results of semi-structured interviews on how people view the conversation between humans and CAs. They found that several social features reported as crucial in human–human conversation, such as understanding and common ground, trust, active listenership, and humor, are not listed as required for human–CA conversations. CA conversations are described almost exclusively by transactional and utilitarian terms. However, this view of CAs is not satisfactory in domains that require the user to engage and form an emotional bond with the CA.

Yand et al. [98] argue that understanding users’ affective experience is crucial to the design of compelling CAs. To elaborate on this claim, they surveyed 171 CA users of Google assistant and examined the affective responses in four major usage scenarios. In addition, they observed the factors that influence affective responses. They found that the overall experience of the user was positive, with the most salient emotion being interest.

Both pragmatic and hedonic qualities influence affective experience. The factors underlying the pragmatic quality are helpfulness, proactivity, fluidity, seamlessness, and responsiveness. The factors underlying the hedonic quality are comfort in human–machine conversation, the pride of using cutting-edge technology, fun during use, the perception of having a human-like assistant, a concern about privacy, and the fear of causing distraction. In the remainder of this section, several issues are discussed that can assist in establishing a deeper connection between the user and the CA during conversations. The focus is on the following aspects: emotional issues, CA personality, and adaptation to the taste and needs of the user.

### 5.1. Emotional Aspect of Conversations

Emotional understanding and empathy are important abilities for CAs acting in several social domains including healthcare, education, and customer support; however, these abilities are also useful to CAs, in general. Combining emotional awareness with technologies and methods for CAs requires multi-domain knowledge in psychology, artificial intelligence, sociology, and education research.

The challenge in enabling empathy and emotionally adjusted responses is twofold: first, the agent must be able to detect the emotional state of the human; second, it must be able to provide the proper emotional response.

The agent may be able to detect user emotions based on user utterances as well as voice and body language. Emotion detection (ED) is an important branch of sentiment analysis and deals with the extraction and analysis of emotions from text and from audio. Acheampong et al. [99] surveyed models, concepts, and approaches for text-based ED and listed the important datasets available for text-based ED. In addition, they discuss recent ED studies, their results, and their limitations. Allouch et al. [100] concentrate on the problem of emotionally insulting sentences recognized by a CA designed to assist the special needs children with their social interactions. They generated a dataset consisting of insulting and non-insulting sentences and compared the ability of different ML methods in detecting the insulting content. In a related study, Schlesinger et al. [101] focus on race-talk and hate speech. They describe technologies, theories, and experiences that enable the CA to handle race-talk and examine the generative connections between race, technology, conversation, and CAs. Drawing together technological-social interactions involved in race-talk and hate speech, they point out the need of developing generative solutions focusing on this issue.

The challenge of listening to the user and understanding the user’s emotional feelings is considered in Sarder’s [102] thesis work, which studies the issue of conversational-agent development for mental-health intervention. Sarder built an embodied conversational agent with three different levels of backchannel strategies and ran a within-subject study with a convenience sample of 24 participants. He showed that the emotional content recognized in the words of the user increases as the CA listening capabilities increase.

As stated above, the second challenge for a CA with emotional abilities is to provide the appropriate response given the user’s emotional state. The ability to recognize the emotions and feelings of others and replying accordingly is known as empathy, which is a crucial socio-emotional behavior for smooth interpersonal interactions. Therefore, the second emotional challenge is to assimilate empathy into CAs.

Empathy can be verbal and non-verbal. Yalcin [103] suggests that embodied CAs should be equipped with real-time multimodal empathic-interaction capabilities. The empathic framework leverages three hierarchical levels of capabilities to model empathy for CAs. Following the theoretical background on empathic behavior in humans, the embodied CA can express empathy by using facial expressions; gaze, head, and body gestures; as well as verbal responses.

Tellols et al. [104] propose equipping the CA with sentient capacities, using ML technologies. They illustrate their proposal by embedding a virtual tutor in an educational application for children. Their CA has a unique personality, emotional understanding, and needs that the user has to meet. The CA’s needs can be expressed by Maslow’s hierarchy of needs [105]. Tellols et al. tested the two CA versions with 10–12 year-old students and found that the second version, equipped with ML capabilities, displays higher understanding capacity and yields a nearly 100% user satisfaction rate. Emotional effects, as well as properties of the speaking style, can be added to the CA to generate speech that is closer to human dialogue.

Chen et al. [106] proposed a conditional text-generative adversarial network (CTGAN), in which an emotion label is adopted as an input channel to specify the output text. To match the generated text data to the real scene, they designed an automated word-level replacement strategy such that after generating initial texts by CTGAN, they extract keywords from the training texts and replace them in the generated texts.

XiaoIce is a popular social CA, developed in 2014 by Microsoft. Zhou et al. [107] describe the design of XiaoIce as an AI companion with an emotional connection. The XiaoIce design includes the intelligence quotient (IQ), the emotional quotient (EQ), and a culturally sensitive personality. The IQ capacity is achieved by knowledge and memory modeling. The EQ capacity includes two key components: empathy and social skills. Both IQ and EQ are combined in a unique personality. The CA personality is defined as the characteristic set of behaviors, cognition, and emotional patterns that form an individual’s distinctive character. XiaoIce’s developers have designed different personas for XiaoIce to suit the preferences and desires of users in different cultures and regions. By analyzing the XiaoIce online logs, Zhou et al. show that XiaoIce understands user intent, recognizes human feelings, generates appropriate responses, and is capable of establishing a long-term relationship.

Asghar et al. [108] propose three methods to incorporate emotional aspects into encoder–decoder neural-conversation models: affective word embeddings, augmenting affective objectives in the loss function, and incorporating a search for affective responses during text decoding. Affective word embedding, in 3D space, can be performed using a cognitive-engineering affective dictionary. Affective objectives can be augmented in the cross-entropy loss function to generate additional emotional responses. Finally, the CA can be guided to search for effective responses during decoding. Asghar et al. show that incorporating these emotional aspects improves the quality of the CA responses in terms of syntactic coherence, naturalness, and emotional appropriateness.

Zhou et al. [109] explain the range of challenges that exist in addressing the emotion factor in large-scale conversation generation. These include: (i) the difficulty of obtaining high-quality emotion-labeled data since emotion annotation is a subjective task, (ii) the need to balance grammar and emotion in expressions, and (iii) the challenge of embedding emotion information. To express emotion naturally and coherently in a sentence, they designed a seq2seq generation model equipped with new mechanisms for emotion-expression generation.

To summarize, considering that the user’s emotional experience and engagement are of great importance in various social and health domains, several studies suggest methods to recognize user’s emotional state to provide an appropriate empathic response. The emotional awareness of CAs can make the user more satisfied and can yield longer and meaningful human–CA conversations.

### 5.2. The Effect of CA Personality

Recent studies have observed that adding personality aspects and human-like characteristics to the conversation may strengthen the connection of the user with the CA. In particular, in the mental-health-care domain, such CAs can elicit higher engagement from humans during the therapeutic process.

Chavesa and Gerosa [110] surveyed 56 studies from various domains to understand how social characteristics in CAs benefit human–CA interactions. They defined eleven social characteristics: proactivity, conscientiousness, communicability, damage control, thoroughness, manners, moral agency, emotional intelligence, personalization, identity, and personality, further grouping them into three social categories: conversational intelligence, social intelligence, and personification. They showed that certain characteristics, such as moral agency and communicability are influenced by the domain, while others, such as manners and damage control, are more generally applicable. They further point out that social-science theories, such as the cooperative principle and mind-perception theories, can contribute to the design of CAs with social characteristics.

Zhang et al. [111] proposed endowing CAs with a profile of a configurable, yet persistent, persona to make them more engaging. This profile is encoded by multiple sentences of textual description. To train the CAs on personal topics, they present a new dialogue dataset consisting of 164,356 utterances between crowd workers who were asked to chat naturally to get to know each other during the conversation.

Inspired by the vision of human-like interactions of conversational agents, Volkel et al. [112] examine the important features of a CA’s personality. They used various sources to examine the main adjectives used by CAs, including an online survey, an interaction task in the lab, and a text analysis of 30,000 online reviews of CAs. They aggregated the results into a set of 349 adjectives, which were rated by 744 people in an online survey. A factor analysis revealed that the commonly used big-five model for human personality [113] does not adequately describe the CA personality. As an initial step in developing a personality model, Vokel et al. proposed an alternative set of main features to be applied to the design of CA personalities.

Feine et al. [114] observed the process of how a social cue evolves into a social signal and subsequently triggers a social reaction. Using the theory of interpersonal communication [115], they identified a taxonomy of social cues of ECAs and classified the social cues into four major categories and ten sub-categories. The four major categories were: verbal, visual, auditory, and invisible. They evaluated the mapping between the identified social cues and the categories using a card-sorting approach.

The effect of ECA personas and cues on user engagement was studied by Liao and He [116]. In their experiment, participants were randomly assigned to racial-mirroring ECAs, non-mirroring ECAs, or control groups. After interacting with the ECA, participants completed a survey assessing their perception and evaluation of the agent. Liao and He demonstrated that racial mirroring has a positive influence on the user’s perceived interpersonal closeness with the agent; the participants interacting with mirroring ECAs reported a higher level of satisfaction, a higher desire to continue interacting with the agent, and predicted a closer future relationship. In addition, people were significantly more likely to select same-race agent personas when they were given an opportunity to customize the ECA.

Go and Sundar [117] tested the distinct and combined effects of three types of cues that potentially enhance the humanness of chat agents: human-like visual cues, the use of human names or identities, and the use of human language. For these three factors, the authors examined how interactions among these cues influence psychological, attitudinal, and behavioral outcomes. Their experimental results indicate that CA interactivity is an important factor in determining psychological, attitudinal, and behavioral outcomes, while the identity cue turns out to be a key factor in eliciting certain expectations regarding CA’s performance in conversation. However, message interactivity can compensate for the impersonal CA nature.

A good open-domain CA should be able to seamlessly blend all its skills, including the ability to be engaging, knowledgeable, and empathetic into one conversational flow. Smith et al. [118] present a method for training a CA with blended skills and testing it. They show that existing single-skill tasks can effectively be combined to obtain a model that blends all skills into a single CA. To preclude unwanted biases when selecting the skill, fine-tuning was done on the blended data.

### 5.3. Personalized CAs and their Effect on Human Engagements

In addition to possessing empathy, persona, and knowledge, the ability of the CA to adapt itself to the user’s taste and needs is also important in engaging the user.

The studies described in this section are related to personalized CAs that adapt themselves to particular users to increase user satisfaction. However, adaptation may come at the cost of a loss in user privacy, which, if observed by the user, may limit the user’s spontaneity in conversation. The effect of users limiting their conversation, upon detecting that the CA is collecting private information to adapt, was reported by [119].

A psycholinguistic characteristic of young adults interacting with a CA is to discuss daily-scheduling concerns and stress levels. Ferland and Koutstaal performed a linguistic analysis that presents the slightly paradoxical effect of reduced user engagement when a conversational agent explicitly discloses information on its user model to the user. They conclude that overt user models may discourage users from self-disclosure and participation in an information-rich spontaneous conversation.

Nevertheless, in task-oriented domains as well as educational domains, adaptation to the user’s abilities and skills may assist the CA to be more effective and may result in higher user satisfaction. Carfora et al. [120] envisage goal-oriented agents whose policies take into consideration the psychological features of the user to deliver personalized and more effective messages. They built a probabilistic predictor based on the theory of planned behavior [121] and a psycho-social model of reference and implemented it by a dynamic Bayesian network.

The smart-learning environment may involve task assignments adapted to the learner’s abilities [122], smart hints and feedbacks [123], smart guidance during the learning process [124], and personalized conversational agents who assist in the learning process [125].

In the healthcare domain, Mandy [126], a primary-care CA created to assist healthcare staff by automating the patient-intake process, provides personalized intake service to patients by understanding their symptom descriptions and generating corresponding questions during the intake interview.

Schuetzler et al. [127] focused on the effect of improving the social presence of CAs by enhancing their responsiveness and embodiment. Responsiveness is the ability of the agent to provide responses contingent on user messages, and embodiment is the visual representation of the agent. In particular, they examined the influence of CA responsiveness and embodiment on the answers people give in response to sensitive and non-sensitive questions. They found that CA responsiveness increases socially desirable responses to sensitive questions.

**Figure 8:**
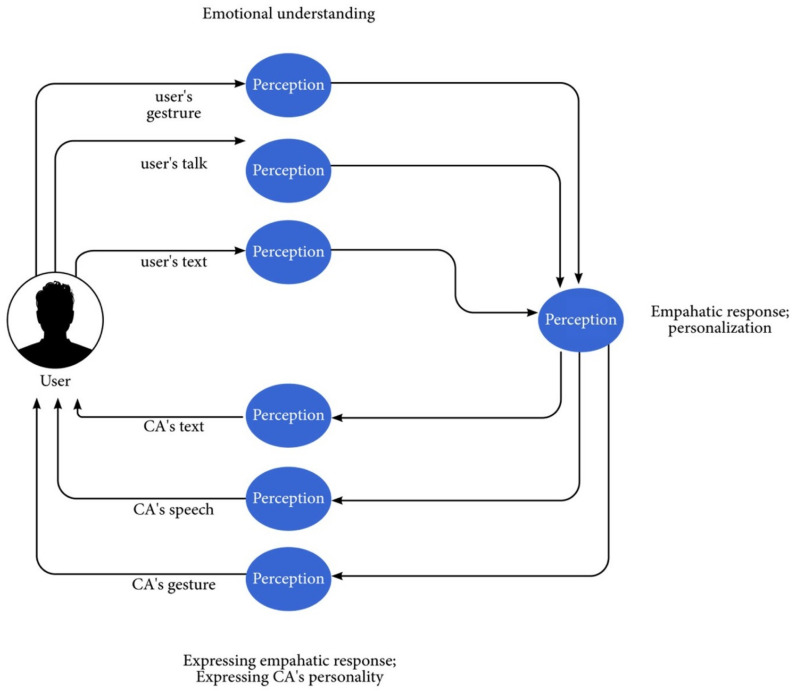
Human-related aspects of the CA: emotion sensitivity, personality expression, and adaptation to the user’s taste and needs.

Figure 8 presents an overview of the human-related issues discussed in this section. Each challenge is associated with the appropriate CA component expected to assume the most responsibility for that challenge. Understanding the user’s emotional state is mostly a challenge of the ASR, NLU, and perception components; the dialogue manager decides on how to provide an appropriate empathic response; the NLG, the gesture generator, and the text-to-speech components are responsible for generating empathy in verbal and non-verbal responses; the personality of the CA is expressed by the response generators including the text-generator, the speech-generator, and the gesture-generator components; and adaptation of the CA to the user’s taste and needs is the responsibility of the dialogue manager.

## 6. Goals and Applications of Conversational Agents

### 6.1. Personal Assistants and Open-Domain Conversational Agents

The first CA was developed in 1964 by Weizenbaum [19]. It was named ELIZA, and it simulated conversations by using a pattern-matching approach. ELIZA was designed to serve as a psychologist and mimicked certain kinds of natural-language conversation between humans and computers. People mistakenly believed ELIZA to be intelligent enough to comprehend a conversation, and some even became emotionally close to it. In 1972, the psychiatrist Kenneth Colby developed PARRY [128], which is a natural-language program that simulates the thinking of a paranoid individual. PARRY was developed to train users to detect people at psychological risk.

DeepProbe [129], RubyStar [130], and Meena [2] are recently developed open-domain chatbots. DeepProbe uses a sequence-to-sequence mechanism to satisfy user queries. RubyStar combines ML models and template- and rule-based responses; it uses topic detection, engagement monitoring, and context tracking. Meena CA is trained end-to-end on data mined and filtered from conversations on social media.

Currently, mobile devices and smart speakers are equipped with powerful agents such as Siri, Cortana, Alexa, and Google Assistant, offering support for a variety of tasks such as question answering, information retrieval, scheduling meetings, sending messages, and controlling smart home devices [10,131]. These assistants constantly listen to hear a wake-up keyword, for example, “Okay Google”, “Alexa”, etc. Once a wake-up keyword is said, the assistant records the user’s command and sends it to a server. The server translates the voice command to text by using an ASR component that parses the text using a parser and uses a natural-language-understanding component to determine the appropriate response or action to be taken by the assistant. For example, a simple query “How are you today?” may be followed by an answer “I’m fine; thank you.” A more-sophisticated question, such as “How many types of mammals are there?” may invoke a web-search that results in an answer such as “There are 6000 different species of mammals”. Commands requesting turning on the lights, setting the temperature of an air conditioner, playing a specific song, or ordering a product are executed accordingly.

Current virtual assistants have several drawbacks. First, they require a steady internet connection. Second, while they usually support multiple languages, they are far from supporting all languages used world-wide. In addition, virtual assistants that order products or book hotels and flights may cause unintentional expenses, e.g., when the user is a child. Misinterpretation may cause the virtual assistant to send an unwanted message. This may be harmful if the wrong message is sent to the wrong person or if a conversation is unintentionally recorded and sent to the wrong person. A virtual assistant may also enable the installation of malware. Misinterpretations may also cause the accidental turning off of the heating in a house with a baby, which may have devastating consequences. Finally, the use of virtual assistants may raise serious privacy concerns, as the user audio is recorded and sent to a server for processing. This challenge is further discussed in Section 9. Virtual assistants usually collect user information during their operation.

Some virtual assistants give programmers the ability to extend their abilities. For example, Alexa allows programmers to extend her abilities using the Alexa Skill Kit (ASK). Participants in the Alexa Prize challenge developed social chatting skills for Alexa. There are few open-domain CAs that enable a lay user, rather than a programmer, to teach the agent to perform new action sequences or new responses. A learning-by-instruction agent (LIA) [132] uses a combinatory categorial grammar (CCG) semantic parser to transform the semantics of each command to a few terms of primitive executable procedures that define the sensors and effectors of the agent. If the user gives the LIA a natural language command and if the LIA does not know how to execute the command, it will ask the user to explain how to realize the command through a sequence of natural-language steps. Once explained, the LIA can execute the command in the future.

SUGILITE [133] is a programming-by-demonstration (PBD) system that uses the Android’s accessibility API to enable users to create automation on smartphones. In case the user specifies commands that SUGILITE does not know how to execute, it prompts the user to demonstrate the command, records the user’s explanation, and automatically generates a script. Thus, SUGLITE can learn to execute an unrecognized command from a single demonstration.

Safebot is a collaborative chatbot that allows users to teach the agent new responses [134]. Safebot allows the users to identify inappropriate responses, which are then removed from Safebot’s database such that future users are not allowed to teach Safebot responses similar to the ones previously tagged as inappropriate.

KBot [135] is a comprehensive open-access CA that exploits the potential of semantic web technologies, federated databases, and NLU. KBot contributes to a better understanding of user queries in the context of linked data by being able to answer different user queries. It can handle tasks such as conversations in English, social-network conversations, FAQs, and mathematical tasks, using information gathered from multiple sources such as DBpedia, Wikidata, and MyPersonality (http://mypersonality.org, accessed on 10 December 2021) datasets.

Finally, MILABOT [74] is a DRL-based CA, developed for the Amazon Alexa Prize competition. MILABOT is capable of chatting with humans through speech or text. It was trained on crowdsource data and real-world-user interactions.

### 6.2. Educational Applications

Online learning has shown significant growth over recent years, in particular, during the COVID-19 outbreak. Unfortunately, in online learning, teachers and students are distant from each other, and therefore, the connection and interaction between them may be insufficient. This may cause online learning to be less effective.

There have been multiple attempts to enhance online learning by using intelligent tutoring systems (ITS) [136], which are customized, computer-based instruction and feedback methods without human intervention. Many include conversational agents, which can interact with the students in natural language during the learning process.

Paschoal et al. [137] surveyed 101 pedagogical conversational agents. They identified the different educational areas for which conversational agents have been developed, discussed common development techniques for pedagogical CAs, and also surveyed the communication strategies used by pedagogical CAs to interact with students. Some successful CAs that are recently used in the education domain are next described. Sara is a CA to assist students with learning [125]. Sara shows online video lectures and asks questions to ensure that the student has understood the lecture. It offers additional information and explanations if the student’s responses are inaccurate. Sara interacts by voice and text when needed and has a voice-based input mode. It was demonstrated to improve learning in a programming task. A similar CA was developed by Paschoal et al. [138] to support software testing. AutoTutor [139] is a computer tutor that simulates the dialogues and strategies of a human tutor. It presents questions and problems from a curriculum script and, according to the learner’s input, decides which action to perform next (e.g., providing a hint or moving on to the next problem). AutoTutor segments the input from the learner into a sequence of words, to assign alternative syntactic tags to words and the correct syntactic class to a word.

MSRBot is a question-answering CA dedicated to software-related issues [140]. It uses a neural network to classify each speech act into one of five speech-act categories: assertion, wh-question, yes/no question, directive, and response. It extracts useful information from software repositories to answer several common software development/maintenance questions.

Hobert [141] presents the design and evaluation of a chatbot-based tutor to help teach beginner programmers to code in university courses. Hobert’s coding tutor is based on teaching-assistant requirements that appear in the scientific literature. Hobert claims that his chatbot tutor is suited to take over the tasks of teaching assistants when there is no human teaching assistant available.

Similarly, Kloos et al. and Aguirre et al. [142,143] introduced the design and features of a CA for Google Assistant [144] to complement a massive open online course (MOOC) for learning Java. Both studies run several experiments and report that users find the conversational agents to be very useful.

Lin et al. [145] developed Zhorai, a CA that enables children to explore AI algorithms and machine learning. Lin et al. showed that by training an agent, observing its mistakes, and retraining the agent, children were able to understand the agent’s ability to learn, as well as obtaining some level of understanding of the learning algorithms used by it.

Cai et al. [146] introduced MathBot, a rule-based chatbot that explains math concepts, provides practice questions, solves problems, and offers tailored feedback. Using mTurk workers, Mathbot was compared to other baseline methods, such as video tutorials and written material. It was found that students prefer MathBot over other options.

CAs can also be useful in foreign-language learning. Indeed, there have been several recent attempts to develop CAs for that purpose. Duolingo’s chatbot with Mondly as well as Andy are some examples of chatbot applications for language learning [147]. Some virtual assistants, such as Alexa, include extensions that enable the learning of foreign languages [148]. Alexa has the skills to assist in building a vocabulary and handling a conversation in a foreign language. Pham et al. [149] developed English Practice, which is a mobile chatbot application to assist a user in learning new vocabulary and to carry on a conversation. Another CA dedicated to language learning is Lucy [150], an embodied virtual agent, designed to help users to learn vocabulary and grammar and to carry on a conversation.

CAs can also be used to support the administration in educational systems. For example, Hien et al. [151] present FIT-EBot, a chatbot that responds to student questions related to services provided by the education system on behalf of the academic staff. Similarly, Ranoliya et al. [152] introduced a chatbot designed to answer visitor questions at Manipal University. It provides an answer based on a dataset of frequently asked questions (FAQ) using AIML. When a user asks a query, the chatbot searches for a similar question and provides the answer to that question. Another chatbot was developed by Keeheon et al. [153] to provide information in educational systems by answering frequently asked questions The chatbot was successfully used by students and department offices in Underwood International College, Korea.

The authors reported that the use of the chatbot had a positive influence on administrative work in reducing workload.

Discussion-bot [154], developed by Feng et al., provides answers to students’ discussion-board questions using natural language. Given a question, it mines suitable answers from an annotated corpus of archived discussions and course documents and chooses an appropriate response.

#### Special-Needs Education and Assistance

In recent years, researchers have expressed a growing interest in using CAs as well as social robots as a positive intervention for children with special needs [155].

PunkBuddy is a tool that includes a chatbot that helps dyslexic students learn through interaction. The chatbot can advise students on the rules of using punctuation, utilizing the benefits of explicit instruction [156].

Park et al. [157] developed a voice-based virtual agent for children with ADHD to help them in their daily tasks. The agent provides vocal feedback to the child and encourages the child to complete the task (on time). The child reports back to the agent about her/his progress.

Xuan et al. [155] developed a chatbot dedicated to children with autistic spectrum disorder (ASD) to improve their conversation abilities. Their chatbot is intended to arouse the curiosity of children and assist them in understanding the conversation better. The chatbot uses a large question-and-answer corpus. Social-assistance CAs are commonly used to assist children and adults with special needs, and especially children with ASD.

Indeed, several studies have shown that social robots can help improve the social skills of children with ASD [158], and some have indicated that a child with ASD might find it easier to interact with a social robot than with a human teacher [159].

Scassellati et al. [160] developed a social robot to increase the social-communication skills of children with ASD. The robot can move or talk according to a selected task defined by the caregiver. For example, the robot can present a social situation and ask the child what the story character is feeling. They reported that after a one-month deployment, the children with ASD improved their behavior and gained their independence.

Costa et al. [161] introduced QTrobot, a social robot developed to assist children with ASD to focus their attention, imitate positive behavior, and reduce repetitive and stereotyped behaviors. QTrobot converses with the child and plays imitation games with the child. Costa et al. showed that children pay more attention to QTrobot than to a person, imitate the robot as if it is a person, and practice fewer repetitive and stereotyped behaviors with the robot than with the person.

Vanderborght et al. [162] developed Probo, which is a social story-telling robot capable of expressing emotions via facial expressions and gaze. Probo uses stories to teach children with ASD how to react in different situations, such as saying “hello” or “thank you.” Probo also teaches children to share their toys. Vanderborght et al. showed that there are situations where the social performance of autistic children improves when using Probo.

Another known robot developed in the same project is Nao. [163], an embedded CA that has been tested and deployed in several healthcare scenarios, including care homes and schools.

### 6.3. Healthcare Conversational Agents

CAs can potentially play an important role in healthcare. There have been several recent reviews on CAs in this field (see [164,165,166,167]). Each points to challenges in the healthcare area pertaining to efficiency, security, and privacy.

CoachAI is a system that includes a chatbot and a machine-learning model to support a patient’s health activities [168]. The chatbot collects data, sends reminders, and converses with users through text-based, simple, graphical elements to guide the user in health-related issues. The model is based on real-world data provided by a health clinic. The application provides the caregivers with insights on the users and assists with the tracking of user activities and their health conditions.

Daily healthcare can be overwhelming for people with a chronic disease. Neerincx et al. [169] developed a social robot that helps children with diabetes. The robot supports the daily diabetes-management processes, namely, taking pills, shots, and body measurements by conversing with the child.

The Watson assistant for health (Watson Health) is an extension of IBM Watson [170] to the healthcare domain. Watson was originally developed for the Jeopardy challenge. Watson Health [171] is a CA for health support. It uses a text-based natural-language interface. It receives a collection of patient symptoms and produces a list of possible diagnoses. The assistant provides detailed annotation as well as links to supporting medical literature. However, a study conducted by Ross and Swetlitz [172] indicates that, in some cancer cases, Watson Health provided unsafe and incorrect recommendations.

Xu et al. [173] introduced KR-DS, a chatbot for the healthcare domain. KR-DS obtains a set of symptoms from the user, recognizes the bio tags of each word using Bi-LSTM, classifies the intent of each sentence, and finally, provides a diagnosis to the user, in natural language, using a medical-knowledge graph. Experiments show that KR-DS outperforms other state-of-the-art methods in diagnosis accuracy.

Fitzpatrick et al. [174] developed Woebot, a medical voice-based CA for cognitive-behavioral therapy dedicated to nonclinical cases addressing low mood and anxiety. Woebot provides mental-health information, recommends activities for specific mood problems, and handles emergency-support services. The users reported an improvement in their mood after using Woebot.

Edwards et al. [175] introduced Tanya, a graphically embodied female agent that supports breastfeeding. Tanya was deployed in a hospital and was accessible to women after birth. Edwards et al. show that women that interacted with Tanya increased their chance of successful breastfeeding for the first six months.

During the COVID-19 outbreak, people require medical information with respect to the outbreak but cannot obtain the information from medical teams, which are overwhelmed. Yang et al. [176] developed a medical chatbot that can be consulted for COVID19-related issues. The chatbot is trained on two datasets, in English and Chinese, containing conversations between doctors and patients on COVID-19.

Despite all the CAs developed in the field of healthcare, the reception of CAs in this field has not been as positive as expected. Palanica et al. [177] examined the perspectives of practicing medical physicians on the use of healthcare CAs for patients. Their results indicate that many physicians believe that CAs would be most beneficial for scheduling doctor appointments, locating health clinics, and providing medication information. However, most of the physicians believe that CAs cannot effectively take care of patients’ needs or provide detailed diagnosis and treatment. Nadarzynski et al. [178] studied the acceptability of CAs in healthcare from the perspective of the general public. While the participants in the study recognized the potential of CAs in healthcare, they stated that their experience is not satisfactory enough and that they are concerned about security issues. Scholten et al. [179] surveyed several CAs in the field of healthcare. They concluded that while CAs can increase the motivation of patients and promote behavioral change, user needs are many times implicit, and these needs cannot be addressed by CAs.

### 6.4. CAs in the Business Domain

Conversational agents are becoming more and more prominent in a diverse range of applications in the business area. According to Dhanda [180], CAs have reduced costs in organizations by approximately USD 48.3 million in 2018 and are expected to reduce costs by USD 11.5 billion by 2023. See Bavarescoa et al. [181] for a literature review on CAs in the business domain with a focus on machine learning. CAs can be used as customer-service assistants, providing answers to frequently asked questions (FAQs), which is a common task that can be handled by CAs.

The Thomas question-answering chatbot [182] uses artificial-intelligence markup language (AIML) for template-based questions like greetings and general questions and latent semantic analysis (LSA) [182] to answer other related questions. If the chatbot cannot find a relevant answer, it asks the user for a clarification.

Another chatbot in the customer service area is SuperAgent [183], which leverages large-scale and publicly available ecommerce data. Given a user request for information about a specific product, SuperAgent provides relevant information from in-page product descriptions and from ecommerce websites. SuperAgent is provided as an add-on extension to the Microsoft Edge and Google Chrome browsers.

Xu et al. [184] created a chatbot to serve users’ requests on social media (Twitter). The chatbot encourages interaction between users and businesses on social media. The chatbot was trained on nearly one million Twitter conversations between users and agents. Their analysis indicates that over 40% of user requests are emotional and do not intend to seek specific information. They showed that their chatbot, which is based on deep learning, yields a higher BLEU score [185] than that of an information-retrieval-based system.

Yan et al. [186] introduce a chatbot, dedicated to online shopping. The goal is to assist online customers in purchase-related tasks by answering specific questions and searching for a product. They integrate this system into a mobile online shopping application with millions of consumers.

Another chatbot is SamBot [187], which is integrated into Samsung’s website to answer user questions. Its knowledge base includes: Samsung promotion, Samsung product FAQs, and general information related to Samsung (e.g., open hours and branch locations). If a proper answer cannot be found, SamBot generates a random answer. It can also recommend users questions to ask. They show that SamBot is capable of handling Samsung-related questions very well.

Kaghyan et al. [188] reviewed the aspects of business-to-business (B2B) tools including the use of CAs. In their article, they describe several methods and platforms for creating Facebook chatbots that support a business. Detailed descriptions are provided for three chatbot-creation platforms: Chatfuel, ManyChat, and “It’s Alive!” and a comparison was performed with respect to capabilities, strengths, and limitations.

Another use of CAs in the business domain is for negotiation. Lewis et al. [189] demonstrate that it is possible to train end-to-end CAs for negotiation, which is simultaneously a linguistic and a reasoning problem. To achieve this goal, their CAs contain adversarial elements as well as cooperative elements, and the CAs are required to understand, plan, and generate utterances. They collected a dataset of natural-language negotiations between two people to show that their end-to-end neural models successfully imitate human behavior in this domain.

Luo et al. [190] collaborated with a large financial-services company to design a randomized field experiment on the consequences of chatbots hiding or revealing that they are indeed chatbots. They concluded that when the true identity of chatbots is not disclosed, CAs are as effective as proficient workers and four times more effective than inexperienced workers in increasing customer purchases. However, when chatbots disclose their identity before conversation, the purchase rates are reduced by more than 79.7%, and the conversation becomes shorter. Unfortunately, users do not always trust that CAs can provide the required support.

Følstad et al. [191] present an interview study of thirteen users who interact with chatbots in customer support regarding their experience and the factors affecting their trust. The users’ trust was found to be affected by different attributes such as the quality of the CA’s interpretation of the requests and whether the generated text seemed human-like.

Chihsun et al. [192] investigated how users cope with conversations with chatbots that do not make any progress in the field of customer support. They analyzed a three-month conversation log with a chatbot, which was taken by one of the top digital-banking institutions in Taiwan. They found 12 types of conversational non-progress and 10 types of coping strategies on the part of the user.

Abdellatif et al. used Google’s Dialogflow engine [69] to extract the user intent and the entities mentioned in the user input. Their initial training set was collected from a group of software developers and consisted of different ways developers pose similar questions. Additional training data were collected from developers using the initial CA version during a test period.

### 6.5. Influence and Malicious CAs in Social Networks

Several conversational agents are developed for deployment in social networks. These CAs attempt to influence public opinion by persuading specific surfers to take certain actions, consume certain products, or influence political views.

Few internet tutorials [193,194] have been written to guide users in the process of Twitter chatbot development. Adams [195] gives an overview of influence-impersonating CAs, which impersonate a human to influence users on social media. They also state that most impersonator chatbots are very simple and therefore, cannot deceive serious interrogators.

The study of Assenmacher et al. [196] provides insights into markets of influence and malicious chatbots as well as an analysis of freely available software tools, which are used to create them. Similar to Adams, they conclude that current influence chatbots are very simple and, despite the major advances in the literature on CAs, still use very simple automation methods.

Another study in the social chatbot area is that of Kollany [197]. According to Kollany, there is an exponential growth in the number of influence chatbots on Twitter. Kollany gathered data from GitHub on the ways developers collaborate with each other and check social aspects of programming on that platform.

While influence CAs are usually intended only to influence a person’s opinion, some malicious CAs utilize a social network to steal personal and private, information including credit-card and bank-account details, or to spread false information in an attempt to manipulate the stock market [198].

Several studies focus on influence and malicious chatbots acting in social media. Varol et al. [199] used a publicly available dataset of Twitter accounts and manually labeled all users either as humans or influence chatbots. They estimated that 9–15% of active Twitter accounts exhibit influence chatbot behavior. They present a machine learning model to detect influence chatbots on Twitter based on features extracted from the dataset, such as user followers and tweet content and sentiment.

DARPA held a four-week competition in 2015 in which multiple teams competed to detect influence chatbots on Twitter [200]. Out of 7038 Twitter accounts, 39 were labeled by DARPA as influence chatbots. The leading group detected all influence chatbots, using a combination of machine learning techniques along with a user support system.

Lee et al. [201] deployed honeypots in the Twitter social network to identify and analyze content polluters. They investigated the attributes of Twitter users, including user behavior over time, user followers, and user following. They also enumerate features that may assist in identifying content polluters automatically, and they present a classification model. Finally, they show that their model successfully identifies content polluters.

**Figure 9:**
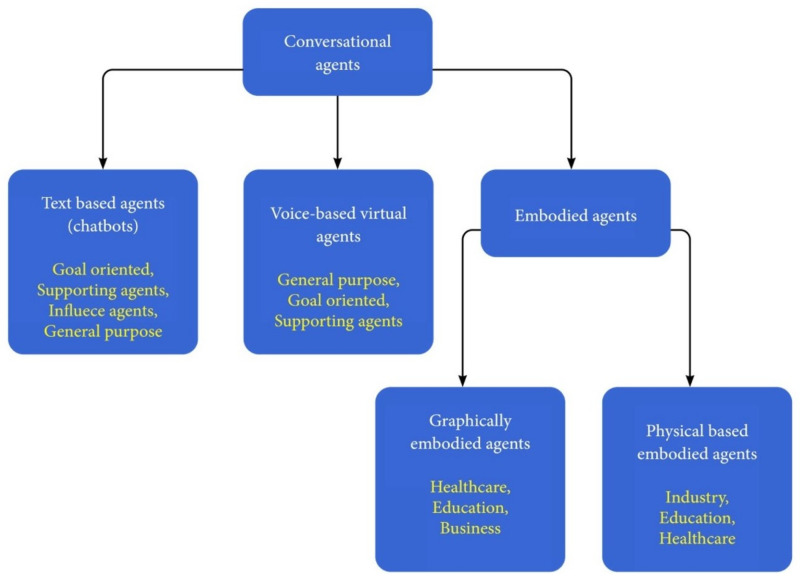
Conversational-agent applications.

To summarize this section, Figure 9 refers to the CA definitions (provided in Figure 1) and, for each type of CA, details the domain of applicability.

## 7. Evaluation Metrics

Three main approaches are used in the literature for evaluating the quality of a conversation agent: human-based evaluation procedures, machine evaluation metrics based on language characteristics, and an ML approach trained on a dataset consisting of human evaluations. The advantages of human evaluation are clear, as humans can evaluate whether the CA responses seem appropriate and resemble responses. However, since human evaluation procedures are expensive, several automatic metrics have been proposed for the evaluation process. Unfortunately, due to the linguistic richness of natural languages and the wide variety of reasonable response options, it is still challenging to achieve accurate and meaningful evaluation when using automatic tools. Therefore, the ML approach tries to benefit from both approaches; on the one side, it is based on human evaluation, and, on the other side, it does not require new implicit costly evaluation methods for each new dialogue situation.

Radziwill and Benton [14] present a literature review of quality issues related to CA development and implementation, focusing on two topics: quality-attributes and quality-assessment approaches. Deriu et al. [202] surveyed the main concepts and methods of CA evaluation. For each type of CA, task-oriented, conversational, and question-answering dialogue systems, they defined the main technologies and the evaluation methods that are appropriate for that type. The requirements of the evaluation methods are stated with respect to automated or partially automated evaluation, repeatability of the results, correlation with human judgment, ability to focus on CA features, and explainability. Finally, Masche and Le [16] divide the different evaluation methods into four classes: qualitative analysis, quantitative analysis, pre/post-test, and CA competition.

In this section, the evaluation methods are divided into three classes, according to the way they are obtained, namely, human-based evaluation, machine-based evaluation, and the ML approach, and some popular evaluation methods are further described for each of these three classes.

### 7.1. Human-Based Evaluation Procedures

As mentioned above, the most accurate method to assess the dialogue quality of a CA is through the score and the qualitative description obtained from humans interacting with the CA. Deriu et al. [202] describe various approaches of human evaluation consisting of lab experiments with users invited to interact with a CA and subsequently asked to fill out a questionnaire; in-field experiments with feedback collected from real users of the CA; and crowdsourcing with crowd workers, either asked to talk to the CA and then rate it or asked to read a produced dialogue and then rate it. The CA rating is based on quality, fluency, appropriateness, and sensibleness.

Venkatesh et al. [18] describe the following metrics to evaluate an open-domain CA: user experience, coherence, engagement, domain coverage, topical depth, and topical diversity. In addition, they propose a unified evaluation strategy, which combines the above metrics into a new evaluation model that correlates well with human judgment. Their unified evaluation strategy was applied throughout the Alexa Prize competition to select the top-performing CAs.

Griol et al. [203] defined a set of specific measures to evaluate the quality of a medically oriented CA. The proposed measures are divided into high-level dialogue features, dialogue style, and cooperativeness. High-level dialogue features evaluate how long the dialogue lasts, how much information is transmitted in individual turns, and how active the dialogue participants are, while dialogue style and cooperativeness features analyze the contents of different speech actions.

To summarize, there are generally three main sources of human-based evaluation: lab sources, real CA users, and crowdsourcing. The information obtained from humans can include: qualitative and quantitative questionnaires, real CA user feedbacks, and dialogue features.

### 7.2. Machine-Evaluation Metrics

Since a high cost is associated with human evaluation, machine-based evaluation or hybrid human-machine-based evaluation are widely used to examine the quality of CAs. Machine-based CA evaluation is challenging due to the lack of an explicit objective for conversation performance measurement. Several studies utilize machine translation-based metrics for CA quality evaluation.

One such metric is the BLEU score [204], a text summarization metric developed for automatic evaluation of machine translation. BLEU takes the geometric mean of the test corpus modified precision scores and multiplies it by an exponential brevity penalty factor. The main component of BLEU is the n-gram precision, which is the proportion of the matched n-grams out of the total number of n-grams in the evaluated translation.

Recall-oriented understudy for gisting evaluation (ROUGE) [205], originally developed for automatic summarization, is also adapted to CA evaluation. Similar to BLEU, ROUGE counts the number of language units, such as n-grams, that appear both in the evaluated summary and in the ideal human-generated summary.

Another popular evaluation metric for machine translation that is applied to CA evaluation is METEOR [206]. METEOR evaluates a translation by counting word-to-word matches between a translation and the reference sentence. If more than one reference is available, the given translation is scored against each reference independently, and the best score is reported.

Liu et al. [207] investigated the usage of the above translation and summarization evaluation metrics for CA. They note that available machine translation metrics assume that valid responses should have significant word overlap with the ground-truth responses. This is a strong assumption for CAs, which exhibit a significant diversity in the space of valid responses. They show that many commonly used metrics for CA evaluation do not correlate strongly with human judgment, and they conclude that there is a need for a new metric that correlates more strongly with human judgment.

### 7.3. Machine-Learning-Based Evaluation

A third approach of CA evaluation is to use ML to predict the human rating of CAs’ dialogues. Lowe et al. [208] present a dialogue-evaluation model called ADEM that learns to predict human-like scores for CA responses, using a dataset of human scores of responses. The human scores were collected using crowd workers that were shown a dialogue context and a candidate response and asked to rate the responses. ADEM is trained by an RNN and, given a response, can successfully predict the appropriateness rating of the response as if it is a human.

Tao et al. [209] propose a routine for evaluating system responses called RUBER. RUBER consists of a Siamese neural network, trained to predict if a pair of context and response are relevant. RUBER is trained using two metrics: a referenced metric measures the similarity between the generated response and the ground-truth response, and an unreferenced metric measures the relatedness between the generated response and the original query. The referenced and unreferenced metrics are combined with heuristic strategies (e.g., averaging) to further improve RUBER’s performance.

Guo et al. [210] propose a topic-based evaluation method on topic breadth, which checks the ability of the CA to talk about a large variety of topics, and topic depth, which checks the ability of the CA to handle a long and cohesive conversation about one topic. A deep average network (DAN) was used to train the topic classifier on a variety of questions and query data, categorized into multiple topics. To summarize, the ML approach of evaluation can be helpful to a wide range of CA researchers and developers as it combines the advantage of human judgment with the advantage of resource saving to rate an unlimited number of CAs and dialogues, utilizing the trained evaluation model.

Table 1 and Table 2 provide the technologies and the evaluation method(s) behind each of the main CAs described in Section 6.

**Figure 10:**
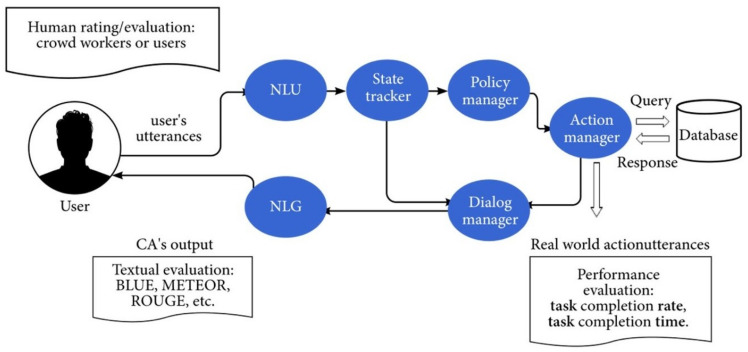
A diagram illustrating the various CA evaluation methods.

Finally, Figure 10 illustrates the various evaluation methods and their relation to each of the relevant components.

## 8. Publicly Available Conversation Datasets

Conversation datasets are used to train machine learning CA models and to test the quality of the CA. In this section some of the existing datasets used in the literature for CA development and CA evaluation are described. Some recent reviews focusing on available conversation datasets are presented next.

Serban et al. [211] review different types of conversations datasets for CAs and categorize them according to the type (text or speech), topics, length (number of dialogs, average number of turns, and number of words), and description.

Keneshloo et al. [212] provide a list of conversational datasets that can be used for sequence-to-sequence models. Some of the databases provided can be helpful for the dialogues generated by conversational agents, and others are related to other domains, such as image and video captioning, computer vision, speech recognition, and synthesis.

Deriu et al. [202] provide another list of available conversation corpora focusing on task related conversations in several domains, such as the restaurant domain and the tourist information domain. They note that question answering dialogue systems can be extracted either from chat logs or from several available literature sources, news, scientific resources, Wikipedia articles, FAQ sites, and even cooking domains.

In the remainder of this section, some of the most useful corpora for conversation understanding, generation, and evaluation are described and classified according to their applications, using the terms defined in Section 2.

### 8.1. Datasets for General Purpose CAs

There are various sources of datasets used for general-purpose dialogues. DailyDialog (http://yanran.li/dailydialog, accessed on 10 December 2021) [213] is a dataset consisting of handwritten texts, manually labeled with communication intention and emotion information. DailyDialog contains multi-turn dialogues, reflecting daily communication on various aspects of daily life. The dialogues in the dataset conform to various common dialogue flows, such as question and answer, bi-turn flows, and multi-turn dialogue-flow patterns reflecting realistic dialogues.

Large amounts of available data on movie reports may also be utilized to build dialogue corpora. The SubTle corpus [214] is designed for general-purpose interaction generation. It is composed of interaction–response pairs, extracted from the OpenSubtitles (http://opus.nlpl.eu, accessed on 10 December 2021) [215,216] movie corpus, which is a multi-language conversation corpus based on movie subtitles. Additional datasets based on movie dialogs are the Movie dialogue dataset (https://www.kaggle.com/abhishek/the-movie-dialog-dataset, accessed on 10 December 2021) [217] and Cornell movie dialogues corpus (https://www.cs.cornell.edu//~cristian/Cornell_Movie-Dialogs_Corpus.html, accessed on 10 December 2021) [218].

Serban et al. [211] consider the advantages and disadvantages of training and evaluating CAs based on artificial datasets, such as datasets extracted from movie manuscripts and audio subtitles. The advantages are as follows: (a) the dialogues resemble human spontaneous language; (b) the dialogues are easy to follow and contain less garbling and repetition; (c) there is a diversity of dialogues, topics, environments, actors, and relationships. This enables creating a more flexible CA, which may talk with various users in different situations while using various interaction patterns. However, since CAs must consider the context to provide accurate responses, Serban et al. state that artificial datasets may have a caveat as they do not provide this context. It should be noted that since dialogues from movies can be too extreme and not reflect real-life dialogues, training and evaluating CAs based on them may lead to undesired behavior on the part of the CAs.

Another source of datasets, for the training and evaluation of CAs, is social media. Many datasets are composed of texts extracted from popular conversation websites and applications, such as Reddit (https://www.reddit.com, accessed on 10 December 2021) and Twitter (https://twitter.com, accessed on 10 December 2021).

Dialogue corpora based on Twitter conversations are developed and used by Li et al. [219], Sordoni et al. [82], Xu et al. [184], and Ritter et al. [220]. Dialogue corpora based on Reddit forums have been developed by several other studies, including the study of Dodge et al. [217], Serban et al. [74], Schrading et al. [221], and recently by Zhang et al. [222]. The dialogue-generation model of PLATO [223] is pretrained on both Twitter and Reddit. The Ubuntu dialogue corpus [224] is based on the Ubuntu chat logs.

Serban et al. [211] note that datasets based on conversations extracted from social media have some significant limitations. Generally, they are noisy, and they may include texts generated by non-human CAs, such as influence agents. Another limitation of Twitter-based datasets is the maximum length of 140 characters per Twitter message. As a result, the Twitter corpus has an enormous number of typos, slang, and abbreviations as well as Twitter-specific structures, such as hashtags. Similar to the issue with artificial datasets, Serben et al. note that dialogues extracted from social media may be missing context. In addition, as stated by Kourosh [225], the use of auto-correction by users of social media may cause an additional layer of complication.

### 8.2. Datasets for Question Answering

Question-answering conversational agents can be trained using publicly available question-and-answer web pages. Zeng et al. [226] surveyed machine-reading-comprehension evaluation and benchmark datasets. They note that the most popular datasets in this category are the Stanford question answering dataset (Squad) versions 1.1 [227] and 2 [228], the CNN/Daily Kail dataset [229], the natural-questions dataset [230], and TriviaQA [231].

The Squad datasets are designed for machine-reading-comprehension training. They consist of more than 100 K questions and answers posed by crowd workers in Wikipedia articles; the answers are citations within Wikipedia articles. The CNN/Daily Mail dataset contains question/answer pairs generated from CNN and Daily Mail articles, published during 2007–2015 for CNN and during 2010–2015 for the Daily Mail.

The natural-questions dataset [230] contains real user questions posted on Google search and answers found on Wikipedia by crowd workers. Each real question may have three types of answers: an associated long answer, which is based on text from a Wikipedia article, a list of short answers, and a yes–no-answer.

Finally, the TriviaQA [231] dataset, designed for machine-reading-comprehension challenges, contains triplets of question–answer-evidence; the evidence aims to ease the answering process. TriviaQA contains relatively complex and challenging questions with syntactic and lexical variability, requiring cross-sentence reasoning in answering TriviaQA questions.

### 8.3. Datasets for Goal-Oriented CAs

The challenge of designing a goal-oriented CA is twofold: the CA should be both effective in NLU and NLG and efficient in helping to solve the common task. Consequently, the task-oriented conversation should take into consideration both aspects. A useful source for obtaining goal-oriented datasets is the dialogue-system-technology challenge (DSTC) [71], which is a yearly challenge started in 2013. Various well-known datasets have been produced and released for every DSTC edition.

The schema-guided-dialogue (SGD) dataset [232], released for DSTC8, contains approximately 23 K annotated multi-domain (bank, media, calendar, travel, and weather), task-oriented dialogues between a human and a virtual assistant. SGD can test state tracking as well as intent prediction, slot filling, and language generation.

MultiWOZ [233] is a tourist-dialogue dataset, annotated with dialogue belief states and dialogue actions. The dialogues in MultiWoz cover seven touristic domains: attractions, hospitals, police, hotels, restaurants, taxis, and trains. Each dialogue in MultiWoz can cover more than one domain.

Taskmaster-1 [234] includes dialogues of the following task-oriented domains: ordering pizza, setting auto-repair appointments, arranging taxi services, ordering movie tickets, ordering coffee drinks, and making restaurant reservations. More than half of the dialogues were created manually, using crowd-workers to compose entire dialogues.

Finally, MultiDoGo [235] is a public human-generated multi-domain dialogue dataset, composed of dialogues created by crowd workers and trained annotators, with a total of over 81K dialogues across six domains. Over 54K of these conversations are annotated for intent classes and slot labels.

For a list of task-related datasets, including DTSC challenges datasets, see Deriu et al. [202].

### 8.4. Datasets for Social Assistance

Social-assistance CAs aim to provide medical, healthcare, mental, or other educational assistance. In these domains, there may exist a privacy issue: information in medical, mental, or educational dialogues is sensitive, and therefore, it is difficult to publish dialogues in a way that would honor the privacy of the participants. Here are some repositories found in these areas.

The first attempt to create a large medical corpus is MedDialog, developed by Zeng et al. [236]. MedDialog is a medical-dialogue dataset that consists of 3.4 M conversations between patients and doctors in Chinese, covering 172 specialties of diseases, and 260 K conversations in English, covering 96 specialties of diseases. Each consultation consists of a description of the patient’s medical condition, followed by a conversation between the patient and the doctor. The data are gathered from Iclinic (iclinic.com) and HealthcareMagic (caremagic.com), which are online healthcare service platforms.

Another health-related dataset was constructed by Yang et al. [176]. Their dataset consists of a collection of conversations in English and Chinese between doctors and patients about COVID-19. The English dataset contains 603 consultations, and the Chinese dataset contains 1088 consultations.

Sharma et al. [237] introduced the task of transforming low-empathy conversational posts into higher-empathy posts. They focus on mental health-related conversations filtered from posts of TalkLife (talklife.com), which is the largest online peer-to-peer support platform for mental-health support. The dataset contains 3.33 M interactions from 1.48 M users posts. The interactions were labeled with empathy measurements using a framework, consisting of three empathy-communication mechanisms: emotional reactions (expressing emotions such as warmth and compassion), interpretations (communicating an understanding, feelings, and experiences), and explorations (improving understanding of the users by exploring feelings and experiences).

Another dataset that can be used for empathic user responses is EmpatheticDialogues (https://github.com/facebookresearch/EmpatheticDialogues, accessed on 10 December 2021) [238]. This dataset consists of 25 K conversations grounded in emotional situations, divided into 32 different emotion categories. The conversations are open-domain and handled between two users, with one responding empathetically to the other. Next, some datasets are described that may be helpful in recognizing emotion, detecting abuse, and generating empathic responses, which are all qualities expected from a CA used for mental and psychological assistance. The emotionally recorded corpus SEMAINE, developed by McKeown et al. [239], is based on recorded dialogues of users talking with an operator who tries to evoke emotional reactions. The corpus includes 20 participants and 100 conversations, all recorded with high-resolution cameras and microphones.

Schrading et al. [221] built a text dataset of domestic abuse, extracted from Reddit. The dataset includes abuse and non-abuse texts. Allouch et al. [240] developed a sentence-level dataset based on 13K sentences related to interactions with children having special needs. The sentences are categorized into four classes: normal sentences, insulting sentences, negative sentences about a different person, or sentences that may indicate a dangerous situation. Chai et al. [241] developed an offensive-response dataset, which consists of 110K input–response chat records in which the response is either appropriate or offensive. These databases can assist in training CAs, allowing the CAs to identify different sensitive situations to respond accordingly.

### 8.5. Educational Datasets

Here, educational datasets that can be helpful for educational CA development are provided.

The BURCHAK dataset [242] is a human–human dialogue dataset for interactive learning of visually grounded word meanings in a foreign language. A learner needs to learn invented words for visual objects (for example, the word ”burchak” for a square) from a tutor. The text-based interactions resemble face-to-face conversations and thus contain many of the linguistic phenomena encountered in spontaneous dialogues. The corpus contains 177 conversations and includes 2454 turns in total.

Wolska et al. [243] annotated a corpus of tutorial dialogues on mathematical-theorem proving. To collect the data, they designed and performed an experiment with a simulated tutorial dialogue system to teach mathematical-theorem proofs. The total corpus comprises 66 sets of dialogue-session logs with 12 turns, on average. There are 1115 sentences in total, of which 393 are student sentences.

Hutzler et al. [244] prepared a bank of questions designed to train high-school students on reading-comprehension skills. The questions were rated by a panel of experts using a set of criteria based on Bloom’s cognitive taxonomy [245].

The CIMA collection [246] includes tutoring dialogues between crowd workers playing the role of students and tutors. The tutoring utterances include educational strategies, such as hint provision and questions asked to check the student’s understanding.

MyPersonality (http://mypersonality.org, accessed on 10 December 2021) is a knowledge base composed of information collected from over six million volunteers on Facebook using a personality questionnaire. MyPersonality is used by KBot [135], a social-media-trained chatbot, to find answers to some questions that cannot be found in other knowledge bases, especially in the psychological and social-science domains.

Table 3 and Table 4 describe the list of datasets available online, which are reviewed in this section. For each dataset, a short description is provided along with some important attributes and the type of conversational agent that uses it, referring to the usage described in Figure 3.

## 9. Conclusions and Open Issues

**Figure 11:**
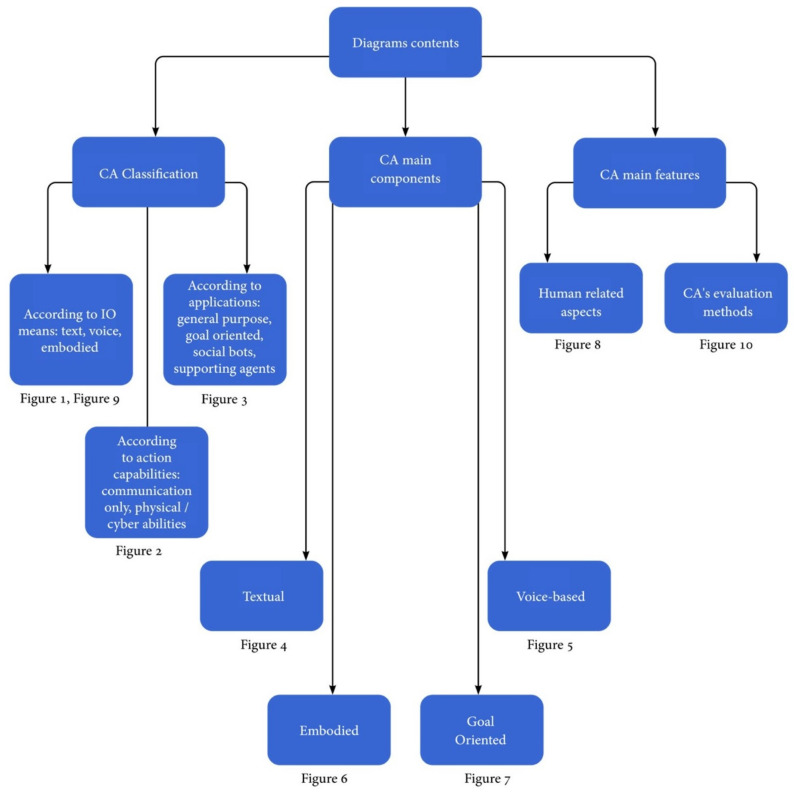
A summary of all diagrams.

In this study, the extensive development of CAs in recent years was reviewed. The leap in the progression of CA development is mostly due to recent advances in deep-learning and big-data technologies. These technologies have led to developments in several domains, such as ASR, NLU, NLG, and emotion-recognition given text, voice, or images, which, combined, allow the creation of a new generation of CAs, with human-like dialogue capabilities. The focus has been on describing the current state-of-the-art technologies developed for conversational agents and various practical applications in which these agents are in use. The survey includes several innovative uses of CAs in various practical areas, including general assistance, task performance, assistance in various social areas, and influence agents, designed to impact the business and public sectors. Figure 11 summarizes the information provided by the different illustration diagrams, which appear in this survey, categorized according to their aims.

There are, however, various additional situations where CAs can be utilized to assist and support people. With state-of-the-art CAs, the most advanced improve themselves based on new data. There are very few CAs, however, that allow humans to teach them additional knowledge and new capabilities or to provide them with the ability to direct their learning process. One of the few systems that can learn directly from humans is commonsense reasoning by instruction (CORGI) [247]. CORGI performs the commonsense reasoning required in applying if-then rules, by initiating a conversation with the user. Another example is Safebot [248], which is taught new responses by the user to avoid learning inappropriate responses. Finally, the learning-by-instruction agent (LIA) [249] asks the user to explain how to execute a new command and associates a sequence of natural-language steps with it. Such systems enable users to fine-tune CAs to adapt them to personal needs and preferences. To further enhance such systems, additional appropriate protocols, algorithms, and rules should be developed and examined.

Another domain where CAs may be useful is in explanatory interactive systems [250,251], which aim to explain to humans the reasons behind decisions made by an automated system. Such explanations are necessary to strengthen the trust between agents and people. CAs may be used to make machine explanations understandable to the human user.

Another area in which CAs are expected to be more prominent is related to consulting a person during his/her conversations. Such a consulting agent would be expected to support people in their daily interactions with other people. The agent is required to model all participants of the conversation to identify their needs in complex social situations to be able to advise them on how to act, talk, or respond in complex social interactions. In our ongoing study [100,240], technology is being developed to assist children with special needs in their daily interaction while monitoring the environment for them.

It should also be emphasized that as CAs become ubiquitous and their ability to provide human-like responses improves, a significant moral question arises: Is there a need to declare the identity of the service or the technical-support representative? Do CAs acting as support or sales agents have the obligation to share their nature with the clients? While studies have revealed that people feel more engaged when conversing with other humans [97], it remains questionable whether maintaining the obscurity of the agent is right, fair, or justified [252].

Another related moral issue arises when considering influential agents. Considering the current state of the technology, any company, party, or ideological movement may develop a CA as a representative to describe its agenda and influence public opinion to garner support for its position. To what extent is such a practice considered moral? Situations where the CA identity is known or hidden should be distinguished, and situations where the company or party is represented by a single CA or by several, hundreds, or even thousands, to create a representation of mass support should be carefully considered and clarified. Surely, using a mass of CAs to influence public opinion seems to be dishonest and unfair, but where is the moral limit?

In addition, given the possibility of such an unfair usage of influence agents, technology should be developed to be able to detect such unfair influence. In Section 6.5, some studies are described that deal with detecting malicious “influence bots”. As the technological ability of such influence bots increases, detecting them becomes more challenging. However, such detection may be crucial, especially when considering extreme groups that may have incentives to utilize such agents for negative purposes.

Several issues arise by the use of assistant agents related to the challenges of protecting user privacy. Mainly, assistant-agent developers must prevent the use of information acquired by the assistance agent by other parties, such as, commercial companies and adversaries. Information-security technologies should be employed to avoid such situations.

To summarize, the rise of CAs and their applications can have a significant influence on our future life. Some of these applications are positive and even crucial, such as health support or social support; others can be beneficial to business and companies; and others should be monitored or even avoided for moral reasons. The limits of fair use of CAs and the technological tools to enforce these limits should be discussed and developed in future research.

## Figures and Tables

**Table 1:** Technologies and evaluation methods for main CA applications: Part A.

Personal Assistants and Open-Domain CAs			
**CA**	**Short Description**	**Main Technology**	**Evaluation Method**
ALICE [48]	a general-purpose chatbot	AIML,	the most human computer
		pattern matching	winner, 2000, 2001, 2004
LSA-bot [50]	ad-hoc implementation	Latent Semantic Analysis	-
	of the LSA framework	(LSA)	
IRIS [51]	example-based	vector space model	success and
	chatbot	cosine similarity metric	failure examples
DeepProbe [129]	an open-domain chatbot	seq-2-seq	AUC scores
	chatbot		
RubyStar [130]	an open-domain chatbot	seq-2-seq, topic detection,	human evaluation
		engagement monitoring,	by the Alexa Prize
		context tracking	evaluation
Siri [1]	Apple’s	CNN,	commercial
	virtual assistant	LSTM	application
Cortana [3]	voice-controlled assistant	NLP, Tellme Networks,	commercial
	for Microsoft windows	Semantic search database	application
Alexa [23]	Amazon voice assistant	NLP, LSTM	commercial
			application
KBot [135]	knowledge	SVM + analytical	F-score, precision,
	chatbot	queries engine	recall, intent classification
MILABOT [74]	speech/text CA	DRL	Amazon Alexa
			Prize competition
Discussion-Bot [154]	question-answering	semantically related	human judges classified
	chatbot	matching, TF-IDF metric	the answers quality
**Goal-Oriented CAs**			
**CA**	**Short Description**	**Main Technology**	**Evaluation Method**
SUGILITE [133]	Programming-by-demonstration	frame-based	a lab study:
	system	dialogue management	task completion time
Safebot [134]	collaborative chatbot	parser+Word2Vec	users’ engagement
LIA [55]	learning by	uses combinatory categorial	speed of task
	instructions agent	grammar (CCG) parser	completeness
**CAs for Social Support**			
**CA**	**Short Description**	**Main Technology**	**Evaluation Method**
ELIZA [19]	the first CA:	pattern matching	people experience
	emulates a psychologist		
XiaoIce [107]	a popular social CA	IQ + EQ + Personality	human rating
Meena [2]	a sensible chatbot	generative chatbot	human evaluation metric
		trained end-to-end on	called Sensibleness and
		social media conversations	Specificity Average (SSA)

**Table 2:** Technologies and evaluation methods for main CA applications: Part B.

Educational CAs			
**CA**	**Short Description**	**Main Technology**	**Evaluation Method**
Sara [125]	student’s assistant	scaffolding strategy	pretest and posttest
			scores of learners
			pro-survey and post-survey
AutoTutor [139]	computer tutor	LSA, pattern-matching	learning gain
		speech act classification	
MSRbot [140]	sofware related Q&A	Dialogflow	effectiveness, efficience
Zhorai [145]	CA for children	NLTK package	accuracy, child’s level
	to explore ML concepts	Website visualizer	of engagement
MathBot [146]	math teaching chatbot	rule based	crowd worker preferences
English Practice [149]	Personal Assistant for	Dialogflow	statistics about
	Mobile Language Learning	platform	real users
Lucy [150]	embodied on-line virtual agent for	ALICE offshoot	demonstrative examples
	language learning		
FIT-EBot [151]	administrative chatbot	DialogFlow	students reports
QTrobot [161]	social robot to assist	bodied humanoid robot	interviews with
	children with ASD		the users
Probo [162]	social robot	compliant actuation systems	children performance
	for children with ASD		
**Healthcare CAs**			
**CA**	**Short Description**	**Main Technology**	**Evaluation Method**
CoachAI [168]	patient’s support	task-oriented finite state	user’s engagement, system
	chatbot	machine (FSM) architecture	accaptance and rating.
Woebot [174]	therapist CA	AI, NLP, empathy engine	users’ reports
Mandy [126]	a primary care CA	NLU, NLG, word2vec	accuracy
Tanya [175]	graphically embodied female		increased
	agent that supports breastfeeding		breastfeeding success
KR-DS [173]	diagnosis chatbot	Bi-LSTM, Deep Q-network	diagnosis accuracy
**Commercial CAs**			
**CA**	**Short Description**	**Main Technology**	**Evaluation Method**
SuperAgent [183]	customer-service chatbot	AIML + LSA	2 customer reviews
SamBot [187]	question-answering CA	AIML	Loebner Prize Competition
			+ user interaction

**Table 3:** Main available datasets for conversational agents—part A.

General-Purpose Datasets				
**Dataset**	**Source**	**Description**	**Size**	**Used for**
DailyDialog [213]	hand written,	daily interactions	13,118 dialogs,	general
	manualy labeled		$\tilde{7}$.9 turns	purpose
[216]	subtitles	interaction–response		purpose
		pairs		
Movie dialogue dataset	movie metadata	OMDb, MovieLens,	3.1 M simulated	Movies QA and
[217]	as knowledge triples	and Reddit	QA pairs	recommendation
Cornell Movie Dialogues	Short conversations	movie metadata	220 K	understanding
Corpus [218]	from film scripts		conversations	linguistic style
Ubuntu dialogue	Ubuntu chat stream	human–human chat	930 K	response
corpus [224]			conversations	generation
**Question-Answering Datasets**				
Squad Version 1.1	questions and answers	$\tilde{1}$00 K questions	100 K q&a	machine reading
[227]	on Wikipedia articles	on Wikipedia articles		comprehension
Squad Version 2	questions and answers	Squad 1.1 +	100 K Q&A +	machine reading
[228]	and additional questions	50 k questions	50 k questions	comprehension
	with no answers	with no answers		
CNN/Daily Mail	queries from the CNN	cont.–query–answer	$\tilde{1}$M stories+	machine reading
comprehension [229]	and Daily Mail websites	triples	associated queries	training dataset
Natural Questions	Google search queries+	Google question+	307,372	training &
dataset [230]	Wikipedia answers	long answer+	training examples	evaluation of
	by crowd workers	short answers		answ. systems
TriviaQA	crowdworkers	question-answer-	95 K quest.-ans.	reading
[231]	questions	evidence triples	pairs + 6 evidence	comprehension
			doc. per quest.	

**Table 4:** Main available datasets for conversational agents—part B.

Datasets for Goal Oriented CAs				
Schema Guided	dialogue simulator+	multi-domain,	20 k	intent prediction,
Dialogue [232]	paid	task-oriented	conversations	lang. generation,
	crowd-workers	human-agent convev.		dialogue tracking
MultiWOZ	turkers working	human-human	10 k dialogues	Task-oriented
[233]			conversations	dialogue modelling
Taskmaster-1	crowd workers	spoken & written	5507 spoken &	dialogue systems
[234]	users and	technical	7708 written	research, dev.
	center operators	dialogs	dialogs	and design
MultiDoGo	crowd workers	human to human,	$\tilde{8}$1 K dialogues	virtual assistants
[235]	paired with	services dialogues	across 6 domains,	development
	trained annotators			
**Datasts for Supporting CAs**				
COVID-19 dialogue	online healthcare	conversations between	603 Eng. +	medical dialogue
dataset [176]	platform	doctors and	1088 Chinese	system
		patients	consultations	systems
MedDialog	medical dialogue	doctors–patients	1.1 M Chinese +	medical dialogue
[236]	platform	conversations	0.3 M English	systems
			dialogues	
SEMAINE	human–human	emotionally coloured	25 recordings,	eliciting non-verbal
[239]	conversation	conversations video	$\tilde{3}$0 min	signals in
	experiment	recordings	long	human-computer
				interactions
EmpatheticDialogues	810 crowd workers	conversations	25 k conversations	recognizing
[238]	select an emotion	grounded in		human’s feelings
	and talk about it	emotional situations		
Offensive response	input–response	input–response	110 K	improve CA
dataset [241]	records from SimSimi	pairs and	chat pairs	abilities
	offensivity annotated	their annotation		
	by crowd workers			
BURCHAK dataset	dialogues of	chat outputs of	177 dialogues	learning
[242]	pairs of participants,	dialogues	2454 turns	visually grounded
	discussing visual			word meanings
	attributes of 9 objects			in a foreign language
The CIMA collection	conversations between	tutoring interactions	2970 tutor	tutoring conversation
[246]	crowd workers playing	and accompanying	responses	based on
	as students and tutors.	responses	to 350 exercises.	a provided strategy.

## Abbreviations

The following abbreviations are used in this manuscript:

AGATA	Automatic generation of IAML from text acquisition
ASD	Autistic spectrum disorder
ASK	Alexa Skills Kit
AI	Artificial intelligence
AIML	Artificial-intelligence Markup Language
ASR	Automatic speech recognition
ASRU	Automatic speech recognition
B2B	Business to business
CA	Conversational agents
CCG	Combinatory categorial grammar
CFG	Context-free grammar
CORGI	Commonsense reasoning by instruction
CTGAN	Conditional text generative adversarial network
DAN	Deep average network
DBN	Dynamic Bayesian network
DNN	Deep neural network
DSTC	Dialogue-state-tracking Challenge
DOAJ	Directory of open-access journals
DRL	Deep reinforcement learning
DRQN	Deep recurrent QNetwork
DSTC	Dialogue system technology challenge
ECA	Embodied conversational agent
ED	Emotion detection
EQ	Emotional quotient
FAQ	Frequently asked questions
GAN	Generative adversarial network
HQ	Hedonic quality
HRED	Hierarchical recurrent encoder–decoder
IoT	Internet of Things
IQ	Intelligence quotient
IR	Information retrieval
IRIS	Informal response interactive system
IS	Information systems
ITS	Intelligent tutoring systems
IVR	Interactive voice response
JA	Joint attention
LD	Linear dichroism
LIA	Learning by instruction agent
LSA	Latent semantic analysis
LSTM	Long short-term memory
MDP	Markov decision process
MDPI	Multidisciplinary Digital Publishing Institute
ML	Machine learning
MMI	Maximum mutual information
MOOC	Massive open online course
MT	Machine translation
NBT	Neural belief tracking
NLG	Natural-language generation
NLP	Natural-language processing
NLU	Natural-language understanding
PCFG	Probabilistic context-free grammar
POS	Part-of-speech
PBD	Programming-by-demonstration
RNN	Recurrent neural network
ROUGE	Recall-oriented understudy for gisting evaluation
SAR	Socially assistive robotics
SCE	Socio-cognitive engineering
SGD	Schema-guided dialogue
SL	Sign language
SQUAD	Stanford question-answering dataset
SSA	Sensibleness and specificity average
SVM	Support vector machine
TF-IDF	Term frequency inverse document frequency
TLA	Three-letter acronym
UX	User experience
